# Multiple-Pathway Synergy Alters Steroidogenesis and Spermatogenesis in Response to an Immunocastration Vaccine in Goat

**DOI:** 10.3390/cells13010006

**Published:** 2023-12-20

**Authors:** Yi Ding, Xunping Jiang, Ling Sun, Yiyu Sha, Zhan Xu, Ahmed Sohail, Guiqiong Liu

**Affiliations:** 1 Key Laboratory of Agricultural Animal Genetics, Breeding and Reproduction of Ministry of Education, Huazhong Agricultural University, Wuhan 430070, China; 2 Key Laboratory of Smart Farming for Agricultural Animals, Huazhong Agricultural University, Wuhan 430070, China; 3 Laboratory of Small Ruminant Genetics, Breeding and Reproduction, College of Animal Science and Technology, Huazhong Agricultural University, Wuhan 430070, China

**Keywords:** immunocastration, DNA vaccine, testis function, goat, transcriptome, whole gene sequencing

## Abstract

Background: Animal reproduction performance is crucial in husbandry. Immunocastrated animals serve as an ideal animal model for studying testicular function. During androgen suppression, the testis undergoes dramatic developmental and structural changes, including the inhibition of hormone secretion and spermatogenesis. Methods: To characterize this process, we investigated the effects of castration using a recombinant *B2L* and *KISS1* DNA vaccine, and then identified functional genes in the testes of Yiling goats using RNA-seq and WGS. The experimental animals were divided into three groups: the PVAX-asd group (control), PBK-asd-immunized group, and surgically castrated group. Results: The results demonstrated that the administration of the recombinant PBK-asd vaccine in goats elicited a significant antibody response, and reduced serum follicle-stimulating hormone (FSH) and luteinizing hormone (LH), resulting in smaller scrotal circumferences and decreased sexual desire compared to the control group. In addition, RNA transcriptome sequencing (RNA-seq) analysis of the testes revealed that the biological processes after immunocastration mainly focused on the regulation of cell matrix adhesion, histone acetylation, negative regulation of developmental processes, apoptosis, and activation of the complement system and the thrombin cascade reaction system. Then, we integrated the whole-genome sequencing and testis transcriptome, and identified several candidate genes (*FGF9*, *FST*, *KIT*, *TH*, *TCP1*, *PLEKHA1*, *TMEM119*, *ESR1*, *TIPARP*, *LEP*) that influence steroidogenesis secretion and spermatogenesis. Conclusions: Multiple pathways and polygenic co-expression participate in the response to castration vaccines, altering hormone secretion and spermatogenesis. Taken together, our atlas of the immunocastration goat testis provides multiple insights into the developmental changes and key factors accompanying androgen suppression, and thus may contribute to understanding the genetic mechanism of testis function. Joint analysis of whole genome sequencing and RNA-seq enables reliable screening of candidate genes, benefiting future genome-assisted breeding of goats.

## 1. Introduction

The goat (*Capra hircus*) is an important domestic species that is widely distributed worldwide and serves as a valuable source of meat, milk, and wool [1]. Consequently, there has been significant interest among researchers in improving the reproductive performance of goats. As a result of long-term artificial selection, their livestock morphology and characteristics have changed greatly with variety diversification, standardized selection schemes, and continuous improvement in reproductive performance [2,3]. The economic traits of livestock are generally subject to positive selection under such directed selective pressures. Selection signals are imprints left on the genome of species by natural and artificial selection over a long period of time, reflecting the influence of selection on breeding. Therefore, the whole genome resequencing (WGS) method is employed to identify potential molecular markers related to quantitative traits in livestock. Utilizing WGS to detect potential positive molecular markers linked to economic traits has emerged as a powerful tool in livestock research.

The reproduction function in mammals is regulated by the complex neuroendocrine interaction of the HPG axis. To study phenotypic differences in reproductive performance, researchers often analyze the transcriptome patterns in reproductive-related tissues. Recently, RNA-seq has been performed using the hypothalamus, pituitary, follicles, ovaries, and testes to screen candidate genes for reproductive performance in various species. However, the direct cause of differences in reproductive performance (ovulation, precocity, sperm development) is the regulation of reproductive hormones [4]. Therefore, this study aims to screen breeding candidate genes by constructing an animal model that reduces the level of key reproductive hormones, and thus affects the expression level of reproduction-related genes. Castration, a process commonly performed in livestock, helps improve meat quality, reduce aggressive behavior, and control unwanted breeding [5]. In this regard, recently, immunocastration targeting the reproductive genes was reported as an animal-friendly, economically efficient method of castration because it can overcome the negative effects of surgical and rubber ring castration [6]. Based on this milieu, immunocastration against gonadotropin-releasing hormone (GnRH) has shown promising results in inhibiting reproduction and has been studied in pigs, cattle, sheep, and other species [7,8,9]. In addition, hypothalamic GnRH secretion is controlled by upriver kisspeptin (*KISS1*) being secreted in the hypothalamus, and the role of kisspeptin as a gatekeeper of GnRH secretion and puberty onset has been revealed [10,11]. In addition to its role in the control of hypothalamic GnRH secretion, the collective data now portray that the *KISS1* gene has a profound effect on the gene and receptor expression on the HPG axis [12,13,14,15]. Given its key role in the modulation of reproduction, we speculate that immunization using kisspeptin vaccine can inhibit fertility in goats. However, it requires an immunomodulator gene to be recognized as a foreign antigen by the animal immune system, due to the fact that kisspeptin is a small molecule produced by the animal body. In this regard, the study suggests that the Orf virus immunodominant gene (*B2L*) can serve as an immunomodulator gene by being recognized as a foreign antigen. The safety and efficacy of the recombinant B2L protein as an adjuvant in veterinary vaccine formulations have been previously demonstrated [16,17]. To the best of our knowledge, the effect of *KISS1* and *B2L* combined immunization on goats has not yet been examined. Whether it can cause kisspeptin immunity in goats while causing Orf virus immunity, and finally achieve the purpose of goat immunocastration, remains unknown. Therefore, the present study was designed to investigate the immunocastration effect of the recombinant ORFV envelope gene (*B2L*) and kisspeptin-54 gene (*KISS1*) DNA vaccine in goats. Moreover, this could serve as an ideal model to screen for candidate genes related to testicular function.

A series of complex biological processes occurred after immunocastration in animals, including retardation of testicular development, hormonal secretion, and spermatogenesis [18]. So far, previous studies have primarily focused on investigating changes in genes and pathways in the testes after testosterone deficiency in rodents [19,20]. However, there are limited empirical data regarding the effect of testosterone deficiency, especially resulting from immunocastration, on testis function in ruminants. In addition, RNA-seq technology provides a powerful tool for detecting dynamic changes in the goat testis. It enables the analysis of RNA expression profiles at the whole transcriptome level, allowing for the identification of gene expression patterns associated with phenotypic changes. Here, we analyzed the testis transcriptomes of 20 goats (2-months-old and 5-months-old immunocastration groups and control groups, respectively) to study the dynamic variety of testis development after immunocastration. In addition, the study also collected data from a total of 93 goats (including 12 wild goats and 81 domestic goats) to detect the selection signal and screen candidate genes affecting reproductive performance. The purpose of this study is: (1) to provide a vaccine candidate for immunocastration in goats; (2) to clarify the dynamic changes in genes in the testis after immunocastration; (3) to combine transcriptomes and whole genomes to screen for testis-related candidate genes.

Overall, this study focused on investigating the dynamic changes in hormones, testis development, and transcriptomes in Yiling goats following immunocastration. By employing whole genome sequencing and RNA-seq techniques on DNA samples and testicular tissue, respectively, the researchers aimed to identify potential candidate genes that might regulate testis function in goats. The findings of this study provide insights into the effects of immunocastration on testis development and gene expression, aiding the development of more effective immunocastration vaccines and deepening our understanding of the testis-related mechanisms in goats.

## 2. Materials and Methods

### 2.1. Experimental Animals and Immunization

The animal experiment was performed in the Yichang Bailihuang Husbandry Co., Ltd. farm (Yichang, Hubei, China). There, 15 health Yiling male goats aged 10 weeks (kid group, 18.20 ± 3.53 kg) and 15 aged 20 weeks (bulk group, 22.23 ± 2.32 kg) were used for this study. All the experimental goats were sheltered in the same environmental conditions. Water was supplied ad libitum and the goats were fed 3–4 kg/day of silage and peanut seedling in a 6:4 ratio supplemented with concentrate (64% corn, 20% soya bean, 8% rice bran, 5% premix, 1.5% salt, and 1.5% soda ash).

The PVAX-*B2L*-kisspeptin-54-asd (PBK-asd) DNA vaccine without an antibiotic resistance gene was constructed according to the procedures as described by Wassie et al., 2019 [21]. Briefly, kisspeptin-54 (*KISS1* gene corresponding to AA 68–121; GenBank accession number NM_002256) and the ORFV isolate MT-05 *B2L* gene (GenBank accession number JN613809.1) were linked using a (G_4_S)_3_ linker and then inserted into a PVAX-asd plasmid. This recombinant plasmid was labeled PVAX-*B2L*-(G_4_S)_3_-kisspeptin-54-asd (PBK-asd). The empty plasmid PVAX-asd was used as a control. The plasmids were transformed into Escherichia coli strains χ6097 and then extracted in a large quantity using the EndoFree Plasmid Maxi Kit (TIANGEN, Beijing, China) according to the instruction manual.

The kid and bulk goats were randomly assigned into 3 treatment groups, respectively: the PVAX-asd group (kid control group and bulk control group), PBK-asd immunization group (kid immunization group and bulk immunization group), and surgical castration group (kid surgical castration group and bulk surgical castration group). Before two weeks prime immunization, surgical castration was performed. The immunization group and the control group were injected with 1 mg/dose of plasmid (PBK-asd or PVAX-asd) intramuscularly. Booster immunization were given at 3-week intervals for a total of 2 booster immunizations.

A blood sample (5 mL) in duplicate for the Yiling goats (*n* = 30) was collected from the external jugular vein at weeks 0, 2, 4, 6, 8, 10, 12, and 14 post-immunization to obtain blood and serum and subsequently stored at −80 °C for later use. At 14 weeks after the initial immunization, all experimental goats were euthanized. During necropsies, tissue specimens were collected in phosphate buffer saline (PBS) for histological analysis and others frozen for RNA extraction for real-time qPCR analysis of the testes.

### 2.2. Experimental Ethics

The animal experiments were carried out in accordance with the guidelines of the Ministry of Science and Technology (Beijing, China: no. 398, 2006) for the care and handling of experimental animals. All the experimental designs and methods involving goats were approved by the Huazhong Agricultural University Animal Care and Use Committee (HZAUGO-2019-006). The utmost efforts were made to minimize the number of animals used, with little to no suffering.

### 2.3. Antibody Detection

The antibody titer in goat serum was detected using indirect enzyme-linked immunosorbent assay (ELISA), and the B2L antigen was coated with the synthetic B2L protein (Bioss, Beijing, China). Donkey anti-goat IgG, HPR (Bioss, Beijing, China) (1:3000) was used as a secondary antibody to detect the bound antibodies. Specific anti-B2L antibody titers were determined using N>μ+2SD, where N is the absorbance (450 nm) of the highest serum dilution, and μ and SD are the mean and standard deviation of negative control samples at the same dilution. 

### 2.4. Hormone, Cytokine Measure and Phenotype Evaluation

The serum testosterone, FSH, and LH hormone levels were determined using enzyme-linked immunosorbent assay (ELISA, Cusabio Biotech Co., Ltd., Wuhan, China), and the operation was carried out according to the instructions. The LH sensitivity of the assay was 0.1 mlU, and within and between batches, the coefficients of variation were <10%. The sensitivity of the FSH assay was 0.1 mlU and the intra- and inter-assay coefficients of variation were 9.8% and 8.3%, respectively. These kits specifically detected the hormones in goat serum without cross-reactivity with other related protein.

The serum cytokines of IL-2, IFN-γ, TNF-α, IL-4, and IL-6 concentration were detected using a goat-specific ELISA kit (Cusabio Biotech Co., Ltd., Wuhan, China). The sensitivity of all the kits is in the range of 1.95–3.9 pg/ML. The intra- and inter-assay coefficients of variation of all kits were <8% and <10%, respectively.

The scrotal circumference was measured at two-week intervals from week 0 of the initial immunization to week 14. A tape measure was used each time to measure the widest part of the scrotum.

The sexuality of the experimental goats was monitored in the last 3 days before slaughter. Four experimental goats were placed in the estrus herd for 30 min to record sniffing the urogenital region of does, vocalization, licking genital organs, foreleg kicking, and butting and mounting the estrus does (female goat). This cycle was repeated until all experimental animals has been monitored.

When the experimental animals were slaughtered, the length, width, and weight of their testes were measured using vernier calipers, and then one testis from each goat was fixed with 10% neutral formalin and stained with hematoxylin and eosin as previously described [22].

### 2.5. Analysis of mRNA Expression and DEGs in Testes

The total RNA was extracted using a Total RNA Kit I (Omega, Mogadore, OH, USA) from the testis, and the quality and concentration were assessed by using a NanoDrop 2000 (Thermo Fisher Scientific, Waltham, MA, USA). Then, the PrimeScriptTM RT Reagent Kit (TaKaRa, Kyoto, Japan) was utilized for the reverse transcription of RNA into cDNA. Next, qPCR was carried and each qPCR reaction consisted of 5 µL of SYBR Green PCR Super Mix, 1 µL of the forward and reverse primers of genes, 1 µL of cDNA, and 3 µL RNase-free ddH_2_O water. The reaction procedure was 95 °C for 30 s, followed by 35 amplification cycles of 95 °C for 5 s, annealing at 57 °C for 30 s, extension at 72 °C for 20 s, and a final extension step of 95 °C for 15 s and 60 °C for 1 min. A reference gene (β-actin) was selected as the control. Differential express genes (DEGs) were chosen at random. The gene sequences used for qRT-PCR came from the NCBI database and specific primers were designed using the Primer software v5 (Supplementary Table S1). The target sequence quantity was normalized to the reference sequence, calculated using 2^−ΔΔCt^, and analyzed using general linear models (GLM).

### 2.6. Transcriptom Profiling

After extracting the total RNA from the collected testicular tissue, it was sent to Biomarker Technologies Co., Ltd. (Beijing, China) for RNA sequencing and library construction. Briefly, the libraries were paired-end sequenced (PE150, the length of reads was approximately 100–150 bp) using the Illumina HiSeq X Ten platform. The FASTQC software v0.11.7 [23] was used to perform quality control on the raw data obtained from the RNA-seq, and the raw data were filtered and trimmed to obtain clean data using the Trimmomatic [24]. Then, the clean data were mapped and assembled using the Hisat2 v2.1.0 and stringTie v1.3.4 software. The goat reference genome sequence used in this experiment is GCA_001704415.1, ftp://ftp.ensembl.org/pub/release94/fasta/capra_hircus/dna/Capra_hircus.ARS1.dna.toplevel.fa.gz (accessed on 20 September 2020). The protein-coding gene annotation file can be downloaded from ftp://ftp.ensembl.org/pub/relee94/gtf/capra_hircus/Capra_hircus.ARS1.94.gtf.gz (accessed on 5 November 2020). The transcriptome raw data of 20 Yiling goat testes have been deposited into the NCBI Sequence Read Archive under BioProject ID PRJNA972266. BioProject and the associated SRA metadata are available at https://dataview.ncbi.nlm.nih.gov/object/PRJNA972266?reviewer=3blrva2ps2l9129isvavf99fcu (accessed on 4 May 2023).

In this study, we used the fragments per kilobase of exon per million fragments mapped reads value (FPKM) to standardize the gene expression level. The DESeq2 package is used for differential gene expression analysis. The screening criteria for differential genes were |fold change| ≥ 2 and *p* < 0.05. The *p* values were adjusted using the Benjamini and Hochberg method. A Volcano plot was created using ggplot2.

Biological process functional annotation was enriched using GO (Gene ontology) (http://geneontology.org/, accessed on 15 December 2020). Since GO ontology does not have goat annotation information, this experiment converted the goat’s Ensembl gene ID into the cow’s homologous gene with Biomart, and then annotated it. The Kyoto Encyclopedia of Genes and Genomes (KEGG) pathway enrichment was analyzed using KOBAS 3.0 (KEGG Orthology-Based Annotation System) (http://kobas.cbi.pku.edu.cn/kobas3/genelist/, accessed on 18 December 2020). Significantly enriched terms were filtrated by a false discovery rate (FDR) < 0.05. The results were visualized via ggplot2.

Weighted gene co-expression network analysis (WGCNA) was constructed using the R package WGCNA (v1.69). The FPKM values of all genes in the treatment group and the control group were inputted for analysis. A power value of 8 was used for network diagram construction using blockwiseConsensusModules.

### 2.7. Whole Genome Resequencing

A total of 93 goats (81 domestic goats and 12 wild goats) were used for the selective signal analysis. Among them, the blood of 35 Yiling goats (Yichang Baililhuang Animal Husbandry Co., Ltd., Yichang, China) was examined by Biomarker Technologies. In addition, the WGS data of 58 goats were collected from the EMBL (http://wwwdev.ebi.ac.uk/, accessed on 5 September 2020) and NCBI databases.

The 81 domestic goats used in this study were of local breeds from all over China. These breeds included 35 Yiling goats, 6 Jianza goats, 6 Longlin goats, 8 Tibet goats, 3 Inner Mongolia cashmere goats, 2 Xinjiang goats, a Wuzhu Muqinbai goat, Liaoning cashmere goat, Yimeng black goat, Chengde goat (non-mountainous), Zhongwei goat, Jining gray goat, Laiwu black goat, Luliang black goat, Caidam goat, Chengdu goat, Jianchang black goat, Shaannan white goat, Anhui white goat, Yaoshan white goat, Matou goat, Xiangdong black goat, Yvdong black goat, Maguan goat (non-mountainous), Guishan goat, Guizhou black goat, and Leizhou goat. Additionally, there were 12 Iranian wild goats distributed in the mountains of Iran. The variety information is shown in Supplementary Table S2.

The DNA library construction and genome resequencing of 35 Yiling goats were completed by Beijing Baimaike Biotechnology Co., Ltd. (Beijing, China). Briefly, high-quality genomic DNA of 1–3 μg was randomly broken into 300–500 bp fragments using ultrasound, and then the fragment DNA was purified and repaired at the end. A tail was added at the 3 ‘end and a sequencing connector was connected to construct the sequencing library. Then, library inspection was carried out, and the qualified libraries were sequenced using the Illumina HiSeq 2000 sequencer to obtain raw reads, with an average sequencing depth of 10×. Furthermore, the raw reads were filtered by removing reads with adapters, low-quality reads, and unknown sequence content of more than 10%. Clean reads were obtained for subsequent analysis. The annotation of the genetic variation information involved the VCFTools and Ensembl Variant Effect Predictor (VEP) tools. The detailed information on the sequencing data of Yiling goats is shown in Supplementary Table S3, and the whole genome resequencing data of the goats used in this study are shown in Supplementary Table S4. The WGS data reported in this study were archived in the NCBI Sequence Read Archive under BioProject ID PRJNA770183. BioProject and the associated SRA metadata are available at https://dataview.ncbi.nlm.nih.gov/object/PRJNA770183?reviewer=u1jr5musplcknk8ttt5gaa2hr9 (accessed on 15 October 2021).

### 2.8. Population Genetic Structure Analysis

Principal component analysis (PCA) was performed using the PCA program in the Plink software package v1.90b6.20 [25] based on all the genetic variations in the autosomes. The ClustalW software was used to calculate the genetic distance between domestic goats and wild goats using the adjacence method. Finally, the Interactive Tree of Life (iTOL) online tool (https://itol.embl.de/, accessed on 27 October 2020) was used to draw the phylogenetic tree. The ADMIXTURE software v1.3.0 was used to predict the ancestor population and hybrid situation of domestic goats and wild goats using the maximum likelihood method. When K = 3, the cross-validation (CV) value was the minimum, which was the optimal number for the ancestor population.

### 2.9. Genome-Wide Selection Signal Analysis

The population differentiation index (fixation index, Fst) between two groups was determined using the window-sliding method (window size 100 KB, slide step length 10 KB). Fst was used to detect the genetic differentiation between domestic and wild goats, and the formula (Al-Mamun et al. 2015) is $F_{st}={(H}_{T}-H_{S})/H_{T}$. HT and HS represent the heterozygous Fst values of the subpopulation and total population, respectively, and the calculation process is realized using the Vcftools software v0.1.17. The R program was used to perform Z-normalized transformation of the Fst values, and the qqMAN program package was used to draw the distribution map of Z(*Fst*) on the whole genome, and the region with Z(*Fst*) > 2.326 was identified as the selected region (*p* < 0.01).

### 2.10. Statistical Analysis

All data in this experiment were presented using mean ± SEM and analyzed using IBM’s SPSS Statistics 20.0 software. The antibody, LH, and FSH data were statistically analyzed using GLM. The Kruskal–Wallis test was used to analyzed the sexual behavior data. The testis size and scrotal circumference data were analyzed using an independent Student’s *t*-test. The power analysis was performed using the pcr.t.est function from the R package. Differences were considered to be statistically significant if *p* < 0.05. The statistical power was set to 0.9.

## 3. Results

### 3.1. Construction and Identification of the Goat Immunocastration Model

To screen and identify the genes affecting goat reproduction, we constructed a low-testosterone goat model via immunocastration. First, a vaccine was constructed and used to explore whether it could successfully achieve immunocastration in goats. The experimental goats were healthy throughout the experiment, and no lesions, edema, or other abnormalities were observed at the injection site, indicating that the vaccine did not cause an allergic reaction and was tolerated by the animals.

### 3.2. Antibody Response

**Figure 1:**
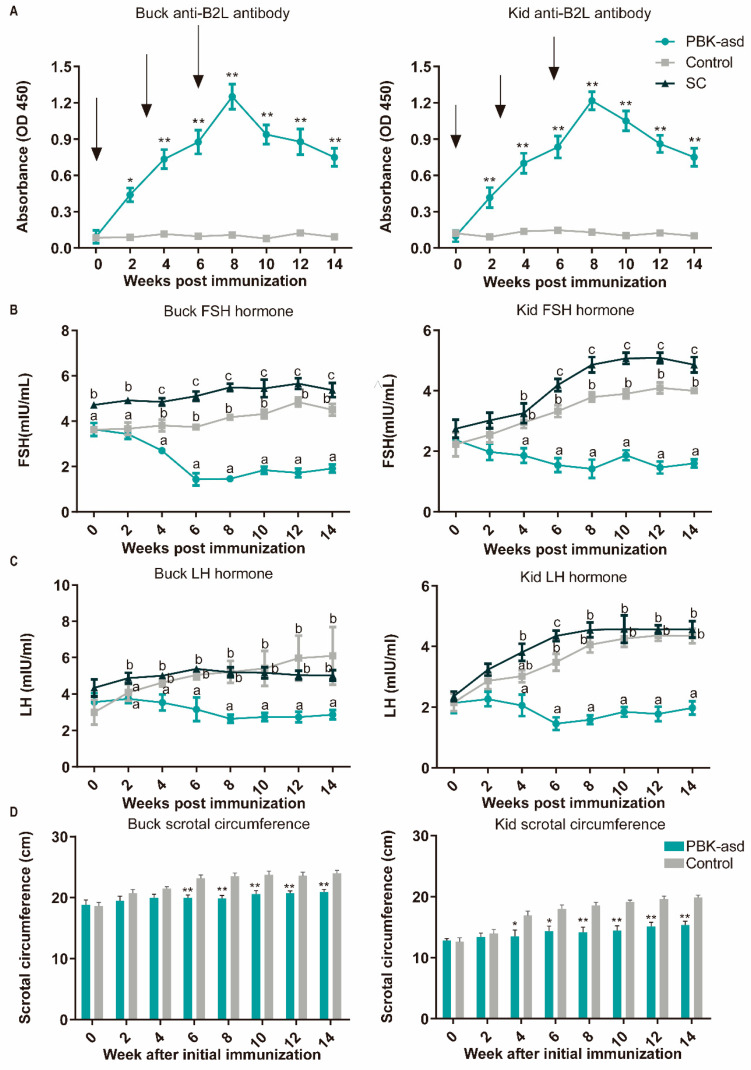

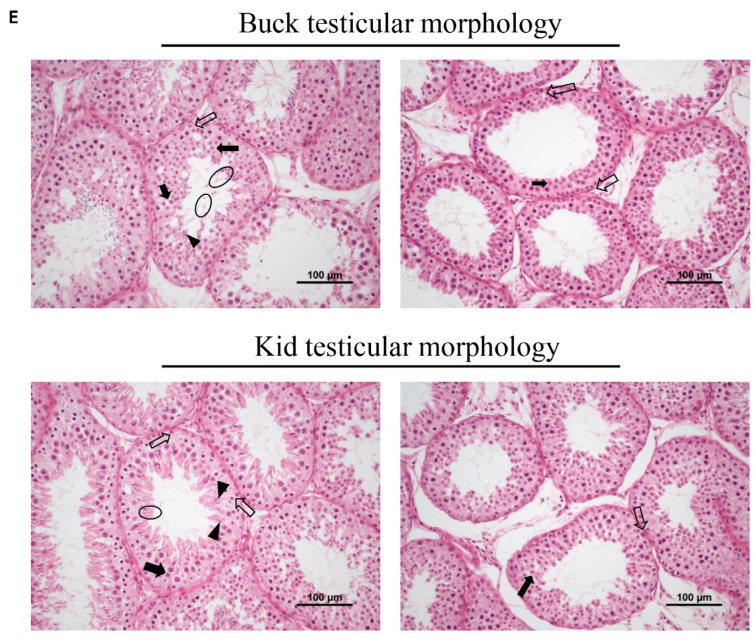
Effect of PBK-asd DNA vaccine on antibodies, hormones, scrotal circumference, and testicular morphology of Yiling goats. The mean ± SEM serum anti-B2L antibody (**A**), FSH (**B**), and LH hormone (**C**) concentrations in buck and kid goats immunized with PBK-asd (*n* = 5), surgically castrated goats (*n* = 5), and control group (*n* = 5), respectively. The treatment was given intramuscularly. Different superscript letter among weeks within a treatment group indicates significant difference at *p* < 0.05. The arrows at the top of the figure indicate vaccination time. * represented *p* < 0.05, ** represented *p* < 0.01. (**D**) The mean ± SEM of scrotal circumference of control (*n* = 5) and PBK-asd-immunized (*n* = 5) buck and kid Yiling goats, respectively. It was measured biweekly from initial immunization to 14 weeks after immunization. * represented *p* < 0.05, ** represented *p* < 0.01. (**E**) Histological analysis of testicular tissue from control goat (**left**) and PBK-asd-immunized goat (**right**) stained with hematoxylin and eosin. The hollow arrow, solid arrow, arrowhead, and circle indicate the spermatogonium, spermatocytes, spermatid, and spermatozoa, respectively. The magnification power is 20× and the scale bar represents 100 μm.

With the aim of determining the antibody response of the experimental goats against the ORFV envelope protein (*B2L*), serum collected from the experimental goats was subjected to ELISA analysis. Interestingly, compared to the control group, an anti-*B2L* response was evident in the immunized goats at two weeks after initial immunization and remained a significant difference to the end of the experiment both in the buck and kid groups (Figure 1A). For both the kid and buck goats, booster immunizations were performed at three and six weeks after initial immunization, and the serum anti-*B2L* concentration in the PBK-asd group reached a peak after the second booster immunization (8th week after initial immunization).

### 3.3. Reproductive Hormone Measurement

Follicle-stimulating hormone (FSH), luteinizing hormone (LH), and testosterone (T) are secreted for the maintenance of spermatogenesis and the manifestation of sexual behavior, and therefore they are a good indicator of fertility. In both the buck and kid goats, at the start of the experiment, there was no significant difference between the PBK-asd and control groups in terms of serum FSH and LH hormone levels (Figure 1B,C). However, surgical castration significantly increased the FSH and LH concentration rapidly after castration and also led to a rapid decline in testosterone levels. A significant difference between the PBK-asd-immunized and control groups was noticed at the fourth week after initial immunization in terms of the serum FSH and LH hormone concentration in buck goats. However, in the kid groups, LH declined as the weeks progressed and had a significant difference until the sixth week from the control. Similarly, in the kid goat group, a gradual decline in testosterone levels was observed starting from the sixth week, with a significant decrease reached by the eighth week. Subsequently, these levels remained consistently low until the end of the trial (Supplementary Figure S1). Our results showed that the serum FSH, LH, and testosterone hormone concentrations of the immunized group were gradually reduced and maintained at low levels.

### 3.4. Sexual Behavior and Scrotal Circumference

To evaluate the effect of PBK-asd immunization on the libido behaviors of goats, sexual behaviors were recorded in the last three consecutive days before slaughter, and the data are presented in Table 1 and Table 2. Since we did not find a significant effect in examining the day-to-treatment interactions, we combined the data from the three test periods. As expected, surgical castration reduced sexual behaviors compared with the control group (*p* < 0.05). The sexual behaviors of the PBK-asd-immunized goats were comparable to the surgical castration group, and intriguingly significantly lower than the control groups of both buck and kid goats (*p* < 0.05).

The data obtained from the scrotal circumference measurement of the PBK-asd and control group goats are presented in Figure 1D. In this study, we found that from the fourth week of initial immunization to the end of the experiment, the scrotal circumference of the vaccinated kid goats was significantly lower than that of the control group (*p* < 0.05), and the immunized buck goats were relatively delayed and showed significant differences at the sixth week (*p* < 0.05).

### 3.5. Testicular Size and Morphology

The testicular weight, length, and width were measured at 14 weeks after the initial immunization, and it was found that the testicular size of the PBK-asd group was significantly lower than that of the control group for both buck and kid goats (*p* < 0.05) (Table 3 and Table 4). The power analysis revealed that the effect size in each group exceeded 2, indicating a substantial variation in the effects observed across different groups.

The testicular histology of the experimental goats was examined using hematoxylin and eosin staining (Figure 1E). The results showed that the PBK-asd immunization destroyed spermatogenesis, which is manifested by a decrease in spermatogonia and sperm cells, with a lack of spermatid and sperm cells in the testicular tissue of the inoculated goats (Figure 1E). In contrast, in the control goats, the spermatogonia, spermatocytes, and spermatids were dense, and sperm cells were also evident (Figure 1E).

### 3.6. Antigen-Specific Cytokine Production

**Figure 2:**
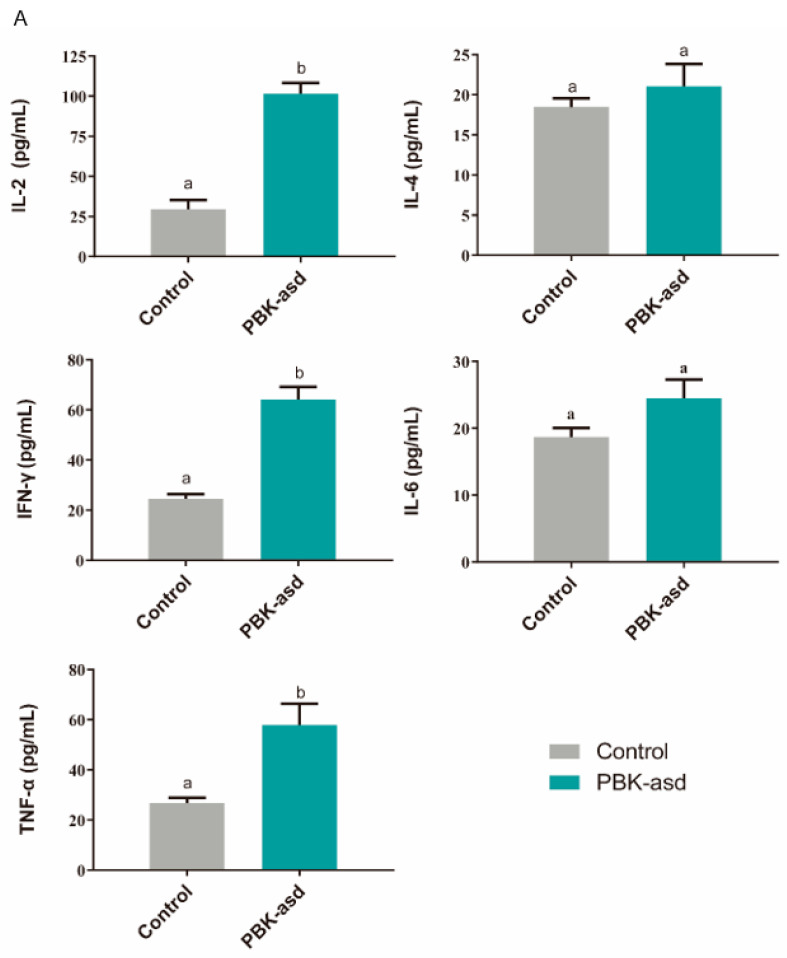

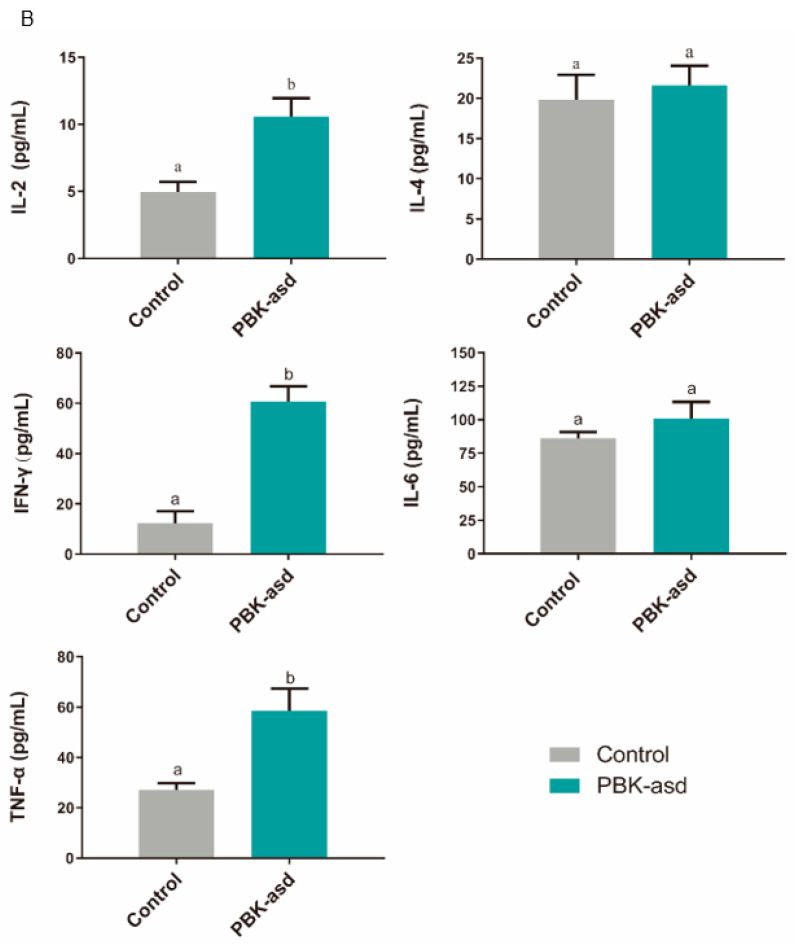
The mean ± SEM of serum cytokines in control group (*n* = 5) and PBK-asd-immunized (*n* = 5) buck (**A**) and kid (**B**) Yiling goats, respectively, detected at eight weeks. Different superscript letters represent a significant difference at *p* < 0.05.

Further, the T-helper 1 (IL-2, IFN-γ, TNF-α) and T-helper 2 (IL-4, IL-6) marker cytokines were identified to determine the pathway of the body responding to vaccine at eight weeks. The results showed that the concentration of IL-2, IFN-γ, and TNF-α were significantly risen compared to the control group after goat muscle immunization (*p* < 0.05) (Figure 2). However, the difference in the concentration changes in IL-4 and IL-6 was not significant. This suggested that the PBK vaccine induced cellular immunity as mediated by the T-helper 1 cells to perform its function.

### 3.7. Testicular Gene Expression Profiles after Immunocastration

**Figure 3:**
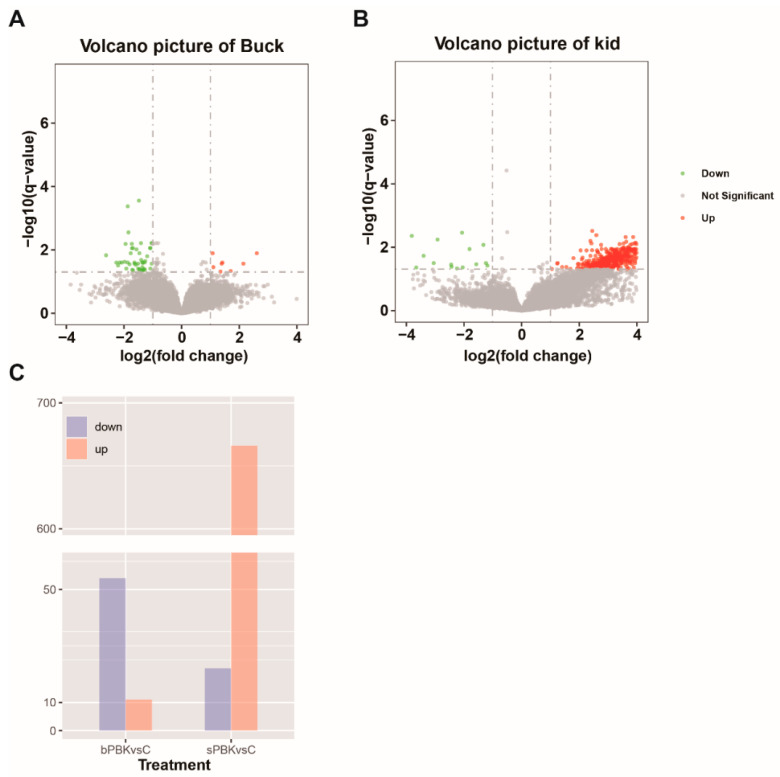
Gene expression profiles of immunocastrated testis. (**A**) Volcano plot showing differentially expressed genes between bulk immunization group and control group. (**B**) Volcano plot showing differentially expressed genes between kid immunization group and kid control group. Red and green dots represent up- and down-regulated genes, respectively while black dots represent the genes without significant differential expression. (**C**) Bar plot presentation number of DEGs between treatment groups: bPBK and sPBK represent buck and kid immunized groups, respectively.

Subsequently, 14 weeks after the initial immunization, the testicular tissue was collected for transcriptome analysis. Then, we performed differential expression analysis of the genes in different groups with *p* < 0.05 and |fold change| ≥ 2 (bulk immunization group vs. bulk control group, kid immunization group vs. kid control group). The analysis found that the bulk immunization group had fewer differential genes (11 up-regulated and 54 down-regulated, Figure 3A), while the kid immunization group differential genes had more changes (Figure 3C). A total of 688 genes changed in the kid goat groups, of which 666 genes were up-regulated and 22 genes were down-regulated (Figure 3B). 

### 3.8. Analysis of Differential Gene Expression in the Buck Immunocastration Group

GO and KEGG enrichment was performed on the differentially expressed genes of the bucks. The results of GO and KEGG analysis for the DEGs are listed in Supplementary Figure S2. The items mainly focused on the negative regulation of cell migration, negative regulation of cell motility, regulation of the cell cycle process, wound healing, neuron projection development, regulation of microtubule cytoskeleton organization, and so on. In addition, the immune process after testis immunization was also enriched in the positive regulation of dendritic cell antigen processing and presentation, negative regulation of T cell antigen processing and presentation, and mucosal immune response. The DEGs were significantly enriched in the KEGG pathway, including those involved in amyotrophic lateral sclerosis (ALS), progesterone-mediated oocyte maturation, the chemokine signaling pathway, axon guidance, focal adhesion, endocytosis, PI3K–Akt signaling pathway, metabolic pathways, and so on.

### 3.9. Functional Analysis of Testis Genes in the Kid Immunocastration Group

**Figure 4:**
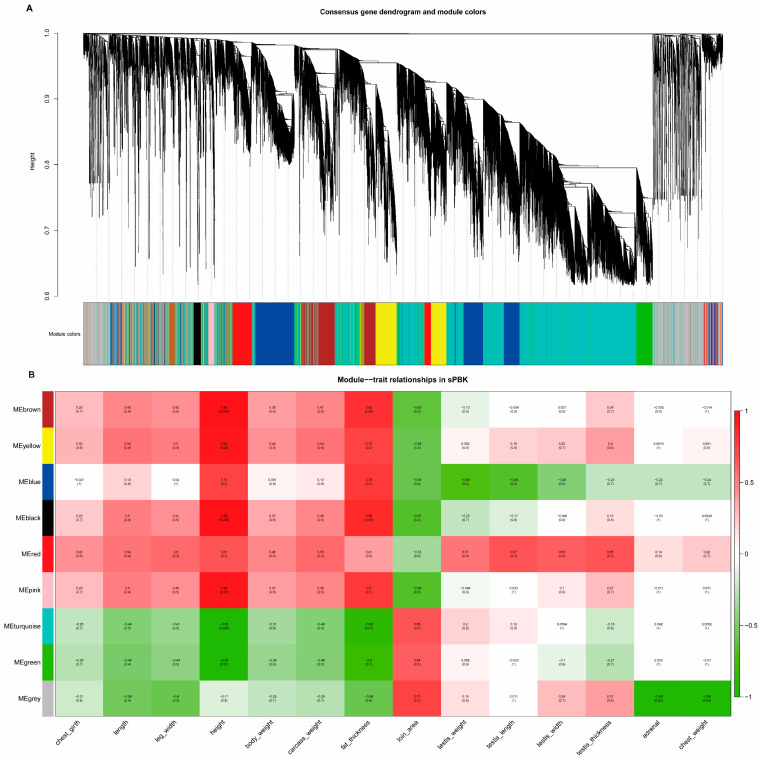
Gene modules identified using WGCNA and relationships of consensus module eigengenes and phenotype traits in the sPBK data. (**A**) Gene dendrogram of all genes of the transcriptome dataset obtained by clustering the dissimilarity based on consensus Topological Overlap with the corresponding module colors indicated by the color row. (**B**) The ME values in kid treatment group were correlated with phenotype traits. Each row in the table corresponds to a consensus module, and each column to a trait. Within each table cell, upper values represent correlation coefficients between ME and the variable, while lower values in brackets correspond to Student’s asymptotic *p*-value.

In order to identify testis-related gene co-expression patterns under different conditions, the RNA-seq data were subjected to WGCNA. In the WGCNA, genes were clustered and a network of eight co-expression modules was identified (Figure 4A). Then, the module eigengene (ME) representing each module was calculated and found. An ME correlation consensus module was used to calculate the correlation with sample traits (body size traits, carcass traits, and testis weight), the results of which were the eigengene significance. The correlation between genes and phenotypes in the kid immunocastration group is shown in Figure 4B. Additionally, the correlation between genes and phenotypes in the control group is shown in Supplementary Figure S3.

As can be seen from Figure 4B, some modules were positively correlated with the immunocastration animal traits, and some were negatively correlated, except for the gray modules (containing non-clustered genes). The ME in the brown and black modules was positively correlated with body height (0.95, 0.95) and fat thickness (0.83, 0.88), but was negatively correlated with loin area (−0.63, −0.67). GO analysis in the black module mainly enriched items for transposition, RNA-mediated and cortisol metabolism, and glucocorticoid biosynthesis and metabolism. This suggested that the black module mainly regulates steroid hormone synthesis, as well as transcriptional and translational modifications. GO items in the brown module were mainly enriched in organic matter metabolic processes, G protein-coupled receptor signaling pathways, positive regulation of cellular processes, skeletal system morphogenesis, and chemical stimuli involving sensory perception. Conversely, the green and blue–green modules were negatively correlated with body height (−0.95, −0.96) and fat thickness (−0.8, −0.86), but positively correlated with loin area (0.64, 0.66). The GO enrichment analysis in the green module mainly focuses on cellular metabolic processes, redox processes, and RNA metabolic processes. The turquoise module was mainly enriched in protein transport along the microtubules, peptide biosynthesis and metabolism, spermatogenesis, and meiosis.

Interestingly, the red and blue modules showed clear opposites in relation to the testis traits (testis weight, length, width, and thickness). Red modules were positively correlated with testis traits, and the GO item results were clustered in the negative regulation of developmental processes, negative regulation of signaling, organization and biogenesis of cellular components, regulation of cell matrix adhesion, anatomical morphogenesis, and so on. However, the ME values of the blue modules were negatively correlated with the testis traits. They were mainly enriched in regulating biomass quality, multicellular development, multicellular organism growth, cellular catabolic processes, and positively regulating enzyme activity.

Taken together, these findings suggested that different module genes acted synergistically to regulate body development and physiological functions after immunocastration. In addition, the mean values of the gene significance (GS) correlation coefficients between genes and testis weight in different modules showed that the blue and red modules had the highest correlation (Supplementary Figure S4A). The enrichment analysis also focused more on regulating growth and developmental processes (Supplementary Figure S4B). This is consistent with the above analysis using ME.

In addition, 10 DEGs were randomly selected for qPCR detection. The results showed that the expression of DEGs in the qRT-PCR assay was consistent with the transcriptome sequencing results (Supplementary Figure S5).

### 3.10. WGS Analysis Identified Positive Genes between Domestic and Wild Goats

#### Population Genetic Structure

**Figure 5:**
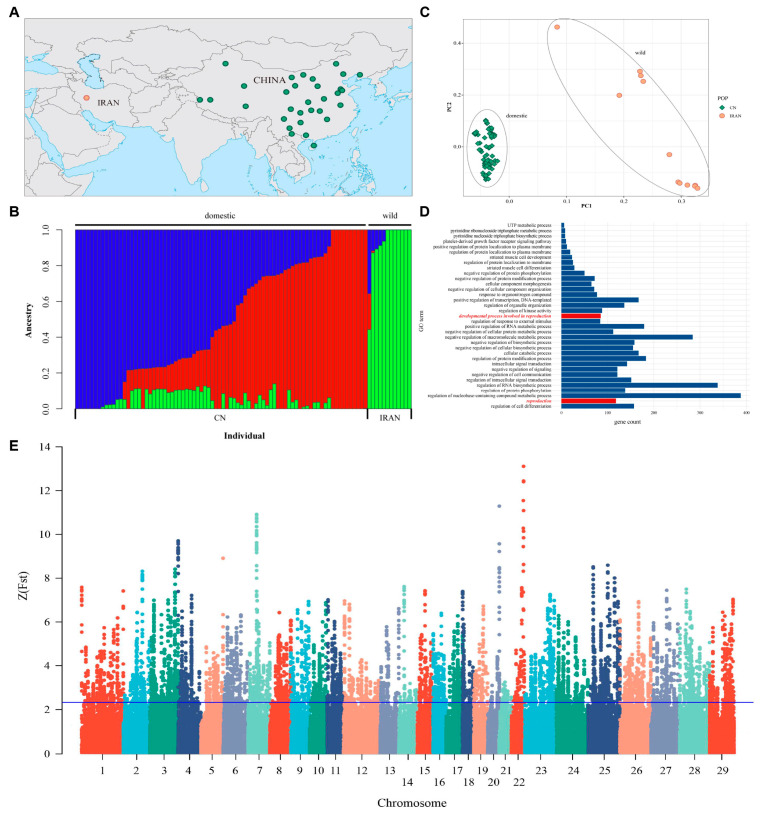
The distribution and kinship of domestic and wild goats in this study, and principal analysis of domestic and wild goats based on SNPs of autosome. (**A**) Distribution of domestic and wild goats in this study. The dots indicate the main distribution areas of different goat populations, not the location of sampling. The green dots represent domestic goats, and the orange dots represent wild goats. (**B**) Ancestry inference based on SNPs of autosome. The CV values of domestic and wild goats were tested when the number of ancestors k was 1 to 5. The CV value was the lowest when k = 3, which indicated that the population was the most reasonable division when they had three ancestors. The x-axis represents each individual, and the y-axis represent the proportion of specific ancestral lineages. A higher proportion of a certain color indicates the higher purity of the blood relationship between the individual and a certain ancestor. (**C**) The consequence shows the genetic distance between domestic and wild populations. The boxes mark the populations of domestic goats, and the dots mark the populations of wild goats. (**D**) Positive selection signals for autosomes 1 to 29 in domestic and wild goats and biological process enrichment of genes located in positive regions. Red marks show the terms related to reproduction. This figure only shows 35 of the 179 terms. (**E**) A sliding window (100 kb window size, 10 kb sliding step size) was used to calculate the *Fst* value between domestic and wild goats, and converted into Z-transformed fixation index. The horizontal line indicates that the window where Z(Fst) ≥ 2.326 is intercepted as an assumed positively selected area.

The geographical distribution of 93 goats in this study is shown in Figure 5A, among which 81 domestic goats were distributed in and around China, and 12 wild goats were distributed in Iran. Then, the genetic relationship between Chinese goats and Iranian wild goats was calculated and shown (Figure 5B). The genetic structure of them can be divided into three lineages, and the lineages of Chinese domestic goat population mainly come from two ancestral populations; there are different degrees of hybridization. The lineages of Iranian ibex are mainly derived from a third ancestral group that clearly distinguishes Chinese goats from Iranian ibex.

Principal component analysis was conducted on Chinese goats and Iranian wild goats (Figure 5C) and the two largest principal components PC1 and PC2 were plotted. The results showed that PC1 separated the wild goat population from the domestic goat population, which formed a tight cluster. In addition, a total of 1215 SNPs were detected in the mitochondrial DNA of Chinese goats and Iranian wild goats. After these SNPs were converted into sequences, the genetic distance was calculated and the phylogenetic tree was constructed using the adjacency method, as shown in Supplementary Figure S6. As can be seen from the figure, Iranian wild goats and Chinese domestic goats were obviously differentiated into two clusters, which was consistent with the results of principal component analysis.

### 3.11. Analysis of Selective Signals on Chromosomes

To screen the genomic regions undergoing positive selection during domestication in Chinese domestic goats, the *Fst* values between Chinese domestic goats and Iranian wild goats were calculated and drawn after standardization (Figure 5E). The window reaching the threshold (*ZFst* ≥ 2.326) was identified as a positive selection region, and a total of 792 candidate selection regions containing 2110 protein-coding genes were screened from 29 autosomes.

GO enrichment analysis was performed on 2110 screened protein-coding genes using the Gene Ontology database (Biological process). A total of 179 significant enrichment items were obtained (Figure 5D). The results showed that the selected genes were mainly enriched in biological processes such as the biological regulation of cellular processes and immune responses. Items related to reproduction were related to the developmental process involved in reproduction, including 111 genes (Table 5). In addition, as supplemented by the literature (Yang et al. 2010 [26]), 22 reproduction-related genes in other items were found, so a total of 133 reproduction-related genes were obtained.

### 3.12. Joint Analysis of WGS and RNA-Seq to Explore the Candidate Genes of Testis Performance in Goats

**Figure 6:**
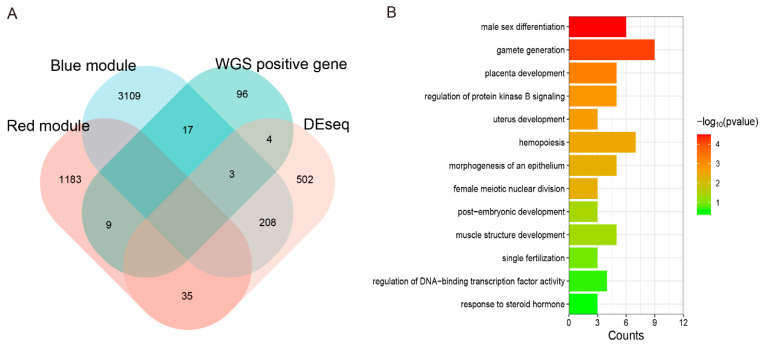
Integrated analysis of whole genomic and transcriptome datasets. (**A**) Venn diagram was made to intersect differential genes, red and blue module genes related to testis growth and development, and positive selection genes in WGS. Genes that overlapped with WGS were singled out. The number in each cell represents the number of genes that intersect. (**B**) GO enrichment of intersected genes from A in biological process.

Based on these results, joint analysis of the WGS and RNA-seq data was performed to further screen candidate genes that may affect the testis function of goats. We intersected differential genes, red and blue module genes related to testis growth and development, with positive selection genes in WGS. Ultimately, 33 genes were obtained (Figure 6A). The detailed information on these genes is listed in Supplementary Table S5. GO enrichment is mainly focused on biological processes such as male sex differentiation, gametogenesis, androgen metabolism progress, and placental development (Figure 6B). Considering the results of the DEGs, Kwithin, and GO pathway, we finally selected 10 genes (*FGF9*, *FST*, *KIT*, *TH*, *TCP1*, *PLEKHA1*, *TMEM119*, *ESR1*, *TIPARP*, *LEP*) as potential candidate genes for the testis function of goats. The details of these candidate genes are listed in Table 6.

## 4. Discussion

### 4.1. Immunocastration Vaccines

Studies have showed that administering kisspeptin centrally can stimulate GnRH and LH secretion in various species [27,28], and mutations in the *KISS1* gene can cause infertility [29]. Building upon these findings, we hypothesized that immunization with the recombinant *B2L* and *KISS1* DNA vaccine may affect the downstream serum secretion of the hypothalamic–pituitary–testis (HPT) axis and cause castration. To test this hypothesis, we measured the serum FSH and LH in experimental goats and found that the levels of FSH and LH in the PBK-asd group were lower than those in the control group. These results suggest that the kisspeptin-specific antibody induced by the PBK-asd recombinant vaccine may neutralize the intrinsic kisspeptin. As a result, the KISS1/GPR54 system becomes impaired, preventing the activation of GnRH secretion by *KISS1*. This leads to reduced GnRH secretion, resulting in decreased pituitary secretion of FSH and LH hormones and ultimately causing infertility. Conversely, in surgically castrated goats, the serum FSH and LH concentration were significantly higher compared to the control group. The elevated serum levels of FSH and LH in the surgically castrated group can be attributed to the loss of the negative feedback effect of testosterone on GnRH secretion [30]. Similar results were noted in bucks and rams immunized against GnRH [31,32,33] and against kisspeptin-54 [34].

Previous studies have shown that immunocastration suppresses sexual behaviors in bucks [33], rams [7,35], and pigs [36]. Similarly, the data from the present study confirm that immunizing goats with a recombinant *B2L* and *KISS1* DNA vaccine can reduce their libido behaviors to levels comparable to surgical castration. The reduction in sexual behaviors in immunized goats may be attributed to a lower serum reproduction hormone concentration.

In addition, the recombinant vaccine used in the current study not only reduced testicular growth but also inhibited spermatogenesis in the immunized goats compared to the age-matched control groups. Furthermore, the histological analysis results showed that the recombinant vaccine not only inhibits testicular growth but also hinders spermatogenesis, as evidenced by no spermatid and sperm cells being present in the testicular tissue of the immunized goats. The suppression effect of testicular growth and spermatogenesis is likely due to the impairment of the KISS1/GPR54 system by anti-kisspeptin antibodies, resulting in a reduction in hormonal secretion on the HPG axis. These results are supported by previous studies showing that KISS1/GPR54 is critical for adolescent seizures and fertility [37,38,39] and target deletion in the *KISS1* gene causes gonadal atrophy [40]. The results are also in line with other studies investigating GnRH vaccines in sheep and goats, which have shown similar inhibitory effects on fertility and sexual behavior [7,41,42]. Therefore, the data from this study show that recombinant *B2L* and *KISS1* DNA vaccines can effectively inhibit fertility and sexual behavior in goats, making them a potential alternative to surgical castration. However, further research is necessary to understand whether the status of their testes is affected by animal growth. Ultrasound is a commonly used and well-tolerated imaging technique that allows for noninvasive observation of normal or pathological conditions in the testes and assessment of their reproductive potential. Therefore, continuous monitoring of the testicular volume and morphology using ultrasound will provide us with more information about the structural changes occurring in the testes.

### 4.2. Changes in Pathways during the Immunocastration Period in the Testis

Animal immunocastration involves a number of major physiological changes, including decreased androgen secretion and the suppression of spermatogenesis, ultimately influencing reproduction function. To better understand this complex process at the molecular levels, a transcriptional map of the testis during immunocastration was generated, which provided a fundamental data resource for multiple analysis modes.

A total of 688 DEGs of kid goats and 65 DEGs of bulk goats was reported, respectively. The DEGs are mainly involved in histone acetylation, the regulation of cell matrix adhesion, the negative regulation of developmental processes, the regulation of cellular metabolism, the regulation of nucleic acid template transcription, apoptosis, and response to stimuli. It has been previously reported that increased levels of H3K9 and H3K18 acetylation will lead to an abnormal morphology with round sperm cells, affecting their further differentiation [43]. In the immunocastration group, abnormal aggregation of acetylation was observed, suggesting that precise regulation of histone acetylation is necessary for spermatogenesis and that abnormal acetylation levels are closely related to male sterility. The MAPK and p53 signaling pathways are well-known regulators of apoptosis [44]. In mammals, the abnormal activity of these pathways can lead to germ cell degeneration and seasonal testicular degeneration [45]. Studies have shown that these pathways are enriched significantly after immunocastration, supporting the hypothesis that vaccine-induced changes in the testis phenotype and internal organization might result from apoptosis. This was consistent with other researchers proposing that apoptosis may be the main regulatory mechanism of spermatogenesis in mammals [46]. Testicular cell-matrix-adherent junctions played an important role in the maturation of Sertoli cells and sperm [47,48,49]. Changes in these junctions may involve the complex regulation of cellular self-renewal, meiosis, and chromatin remodeling in the testes. Multiple kinases in the TGF-β pathway can be activated by a variety of cellular stimuli or toxic stimuli, which in turn affect protein synthesis, metabolism, and substrates of the cell cycle, controlling key cellular processes. During the final stages of spermatogenesis, protamine phosphorylation is essential for the morphological differentiation of haploid sperm into mature sperm. After immunization, the pathway of phosphorylation was significantly enriched and activated compared to the control group. We reasoned that the vaccine might regulate sperm maturation and motility through phosphorylation. Tight junctions between the Sertoli cells in the blood–testis barrier are essential for the transitional maturation of spermatogenic cells during spermatogenesis. Abnormal junctions of Sertoli cells can lead to the obstruction of spermatogenic cell migration in the seminiferous tubules, which may be one of the causes of oligospermia [50]. WNT proteins are secreted morphogens that play a critical role in fundamental developmental processes in various species and organs, causing cytoskeletal remodeling and alterations in cell adhesion and motility. According to previous studies, the above pathways were highly expressed before sexual maturation in goats [51]. However, in this study, the testis samples were collected at 160 days, which is generally considered a sexually mature stage. Therefore, immunocastration may delay the formation of the blood–testis barrier.

The mammalian testis has a specialized microenvironment that prevents the immune system from recognizing specific antigens on germ cells [52]. Disruption of this immune balance can lead to testicular inflammation and male infertility. During the immunocastration period, the MAPK-, TNF-, and Jak-STAT-signaling-pathway-related cytokine genes in the red and blue modules had up-regulated expression, which can induce a variety of intracellular signaling pathways, including those involved in apoptosis, inflammation, and immunity. The complement system, a proteolytic cascade in the plasma, is an important factor in inducing inflammation and subsequent inflammatory responses, and the thrombin cascade, a non-specific defense mechanism against pathogens, was up-regulated in this study. These findings indicated that the transcription levels of immune-related pathways in the testis increased significantly during the immunocastration period, indicating a strong adaptive immune response in the testis, which may affect testicular function.

In addition, studies have also suggested that the vaccine may activate lymphocytes through the MHC-I processing pathway in goats. This was consistent with the detection of cytokines that indicated the significant role played by T-helper 1 cells. MHC-I molecules are mainly involved in scanning for intracellular pathogens and presenting antigens to CD8^+^ T cells, so they are necessary for eliciting cellular immune responses against intracellular pathogens. Based on this information, it is hypothesized that the Orf virus produced by the PBK vaccine in vivo was internalized and then was recognized as an antigen, which will be presented or cross-presented mainly through the MHC-I pathway, with the help of T-helper 1 cells, ultimately causing a CD8^+^ CTL^−^ mediated response to achieve immune protection.

### 4.3. Key Genes Involved in Testis Function Using WGS and RNA-Seq Joint Analysis

In this study, we conducted a comprehensive analysis to identify candidate genes related to testosterone secretion and spermatogenesis in bucks. We utilized a combination of whole genome screening for positive selection genes, differential gene expression analysis in immunocastrated animals, and identification of module genes in the co-expression network. These identified candidate genes can potentially be used to characterize specific functions of the reproductive system and identify some specific molecular breeding targets.

The migration, maintenance, proliferation, and differentiation of germ cells are the main events in the animal testis. A number of studies have shown that *KIT* promotes the proliferation of spermatogonia and inhibits cell apoptosis, and it plays a role in initiating or maintaining meiosis. Heterozygous mice with *KIT* showed lower sperm incidence and smaller seminiferous tubules [53], which aligns with our findings of the impact of *KIT* gene on goat testis function in spermatogenesis. *FST* regulates hormone secretion and spermatogenesis by influencing the activin protein activity. Overexpression of the *FST* gene in mice resulted in a significant decrease in testosterone, while FSH and LH remained unchanged. This suggests that *FST* may function as a local regulator of activin action in gonad development and gametogenesis [54]. In this study, its expression was up-regulated in the immunocastration group, and this may be a cause of hormone secretion and spermatogenesis declining in the testes. *TMEM119* is expressed in the developing testes, sperm cells, and mature sperm, and its knockout can lead to stopping the round spermatid phase [55]. We speculate that *TMEM119*, as a membrane protein, interacts with specific chaperone proteins in Sertoli cells to facilitate cell-to-cell communication, thereby enabling normal sperm differentiation and migration. Sperm–oocyte interaction is one of the most remarkable processes in cell biology. The *TCP1* complex (CCT/TRiC) containing chaperone protein is present on the surface of the capacitive sperm and interacts with the zona pellucida to mediate sperm–oocyte interaction [56]. Therefore, abnormal expression of the *TCP1* gene in the testes is likely to affect the function of mature sperm. In addition, immunocastration can lead to testicular dysfunction, which may result in sexual torsion and significantly impact the expression of sex-differentiated genes. We have identified the *ESR1* gene, which is essential for the development and function of male organs and induces sex reversal in animals [57,58]. In sex differentiation, except for initiating sex-specific signaling cascades, the opposite pathway is inhibited at the same time. The *FGF9* gene was significantly up-regulated and located in the opposite pathway (SOX9/FGF9) which controls gonad development and differentiation and may also contribute to sex reversal when abnormally activated [59].

The synthesis and secretion of steroid hormones is another major and very important function in the testes. In this study, several hormone-related genes were found. The Adhesion junction (AJ) complex is essential for cell–cell adhesion, tissue formation, and signaling. *PLEKHA1*, a component of intercellular AJs, plays a role in interstitial cell differentiation, androgen metabolism, tissue and organ remodeling, and luteinization [60]. Therefore, it may affect testicular function by influencing the formation of interstitial cells and the androgen metabolic pathway. *TIPARP*, also known as *PARP7*, is a single ADP-ribosyltransferase that regulates innate immunity and stem cell pluripotency [61]. It is a direct target gene of androgen receptors (ARs) and directly influences androgen metabolism. It is known that tyrosine hydroxylase (TH) responds to steroid hormones and influences the body’s response to acute stress challenges. Gonadectomy increased TH immunoreactivity in the prefrontal cortex and decreased open field activity in male rats [62]. Its high expression in androgen-deficient testes may contribute to the weak aggression in immune-castrated goats, suggesting a role in modulating immune reactivity. The leptin (LEP) signal of the nutritional status of the body is recognized by the hypothalamus to regulate reproduction. Infertile mice lacking LEP (Lep-/Lep-) have been observed in studies [63]. LEPR, the receptor for LEP, is present at all levels of the HPG axis. *LEP* in immature testes may promote the development of Sertoli cells, Leydig cells, and primordial germ cells, as well as mediate spermatogenesis and testosterone secretion in mature testes. Thus, abnormal expression of *LEP* or its receptor may have a large effect on testicular function. Overall, considering GO terms, DEGs, module genes, positive selection genes, and a literature review, we identified *FGF9*, *FST*, *KIT*, *TH*, *TCP1*, *PLEKHA1*, *TMEM119*, *ESR1*, *TIPARP*, and *LEP* as candidate genes that have the potential to affect the secretion of testosterone and the function of spermatogenesis in bucks, so as to further influence reproductive processes.

## 5. Conclusions

By integrating transcriptome and genome-wide data, multiple pathways and genes were found to be involved in the castration vaccine response, and some candidate genes related to testicular function were identified. A recombinant B2L and KISS1 DNA vaccine, lacking an antibiotic resistance gene, effectively suppresses fertility and sexual behavior in goats by interfering with hormone production on the HPG axis. Using immunocastrated goats as a model, the vaccine was found to affect histone acetylation, regulation of cell matrix adhesion, negative regulation of developmental processes, apoptosis-related pathways, and immune cell infiltration, leading to steroidogenesis disruption and impaired spermatogenesis. A comparison of domestic and wild goat genomes revealed reproductive genes under positive selection. Genes affecting hormone secretion and spermatogenesis, such as *FGF9*, *FST*, *KIT*, *TH*, *TCP1*, *PLEKHA1*, *TMEM119*, *ESR1*, *TIPARP*, and *LEP*, were identified through integrated analysis of WGS and RNA-seq. Overall, multiple pathways and polygenic co-expression participate in the response to castration vaccines, altering hormone secretion and spermatogenesis. The recombinant vaccine has potential for immunocastration in goats and provides insight into using kisspeptin for reproduction regulation. These screening genes offer candidates for further study of testicular function and contribute to future molecular breeding for highly fertile goat breeds.

## Figures and Tables

**Table 1:** The mean ± SEM of sexual behaviors of control (*n* = 5), PBK-asd-immunized (*n* = 5), and surgically castrated (*n* = 5) buck goats recorded over a 30-min interval for the last 3 days of slaughter.

Parameters	Treatment Group		
	Control	PBK-asd	SC
Sniffing	4.8 ± 0.59 ^a^	1.2 ± 0.17 ^b^	0.8 ± 0.17 ^b^
Vocalization	7.4 ± 0.54 ^a^	0.6 ± 0.36 ^b^	0.2 ± 0.17 ^b^
Licking	4 ± 0.56 ^a^	1.0 ± 0.28 ^b^	0.6 ± 0.22 ^b^
Forelimb kicks	2.4 ± 0.22 ^a^	0.4 ± 0.21 ^b^	0. 4 ± 0.22 ^b^
Butting	3. 6 ± 0.61 ^a^	0.6 ± 0.22 ^b^	0 ± 0.0 ^b^
Mounting	5.8 ± 0.82 ^a^	0.4 ± 0.22 ^b^	0.2 ± 0.19 ^b^

Means across a row denoted with different superscript letters differ (*p* < 0.05) when using ANOVA procedures (Kruskal–Wallis test).

**Table 2:** The mean ± SEM of sexual behaviors of control (*n* = 5), PBK-asd immunized (*n* = 5), and surgically castrated (*n* = 5) kid goats recorded over a 30-min interval for the last 3 days of slaughter.

Parameters	Treatment Group		
	Control	PBK-asd	SC
Sniffing	4.0 ± 0.63 ^a^	1.2 ± 0.33 ^a,b^	0.6 ± 0.35 ^b^
Vocalization	7.0 ± 1.02 ^a^	0.6 ± 0.35 ^b^	0 ± 0 ^b^
licking	3.0 ± 0.40 ^a^	0.6 ± 0.22 ^b^	0.4 ± 0.21 ^b^
Forelimb kicks	3.0 ± 0.40 ^a^	0.6 ± 0.36 ^b^	0. 2 ± 0.18 ^b^
Butting	3. 2 ± 0.65 ^a^	0.2 ± 0.18 ^b^	0 ± 0.0 ^b^
Mounting	4.8 ± 0.59 ^a^	0.2 ± 0.18 ^b^	0 ± 0.0 ^b^

Means across a row denoted with different superscript letters differ (*p* < 0.05) when using ANOVA procedures (Kruskal–Wallis test).

**Table 3:** Effect of PBK-asd DNA vaccine on testicular size of Yiling goats.The mean ± SEM testicular weight, length, and width of control (*n* = 5) and PBK-asd-immunized (*n* = 5) buck goats at 14 weeks after initial immunization.

Parameters	Treatment Group		Effect Size
	Control	PBK-asd	
Testis weight (g)	65.54 ± 4.10 ^a^	49.76 ± 2.52 ^b^	2.80
Testis length (cm)	6.37 ± 0.22 ^a^	5.58 ± 0.12 ^b^	2.84
Testis width (cm)	4.84± 0.12 ^a^	4.20 ± 0.09 ^b^	3.03

Means with different superscript letters across a row indicate a significant difference at *p* < 0.05.

**Table 4:** The mean ± SEM testicular weight, length, and width of control (*n* = 5) and PBK-asd-immunized (*n* = 5) kid goats at 14 weeks after initial immunization.

Parameters	Treatment Group		Effect Size
	Control	PBK-asd	
Testis weight (g)	49.38 ± 2.58 ^a^	38.28 ± 1.79 ^b^	2.76
Testis length (cm)	5.97 ± 0.16 ^a^	5.27 ± 0.10 ^b^	2.98
Testis width (cm)	4.14 ± 0.10 ^a^	3.79 ± 0.06 ^b^	2.94

Means with different superscript letters across a row indicate a significant difference at *p* < 0.05.

**Table 5:** GO enrichment results related to the reproduction process.

Item	Number of Gene	Gene Name
Development progress involved in reproduction	80	*VIPAS39*; *DMRT3*; *PLEKHA1*; *TRIP13*; *NUP210L*; *AREG*; *TPGS1*; *GREB1L*; *SENP2*; *ERCC1*; *CYP26B1*; *DPCD*; *FBXW11*; *YTHDC2*; *WASHC5*; *IFT81*; *FST*; *CSF1*; *RXFP2*; *SPINT2*; *ETV2*; *ESR1*; *YY1*; *DNMT3A*; *DMRT2*; *CNTFR*; *PRKDC*; *VDR*; *SYCE3*; *SRD5A2*; *KITLG*; *RHBDD1*; *PANK2*; *KRT8*; *ASH1L*; *PMFBP1*; *MTOR*; *RBM15*; *FGF9*; *RXRA*; *OSR1*; *HERC4*; *STK3*; *SMRP1*; *PDGFRA*; *THEG*; *CCDC36*; *LEP*; *IQCG*; *TTC26*; *TIPARP*; *FOXO3*; *DMRT1*; *GALNTL5*; *LFNG*; *KIT*; *KMT2B*; *PCDH12*; *NOS3*; *ADGRG1*; *CCDC134*; *DEDD*; *MOV10L1*; *KIF18A*; *SCX*; *HYAL3*; *CFAP54*; *BIRC6*; *SFRP2*; *CBX2*; *HSF1*; *FANCA*; *SPEM1*; *CCNB1IP1*; *CYP19A1*; *TMEM119*; *MEI1*; *NUPR1*; *SEPT2*; *IGF2*
Reproduction	31(111)	*OXTR*; *UBAP2L*; *TUBGCP6*; *SPIRE2*; *STAG3*; *SIRT7*; *ARSA*; *SELENOP*; *GGN*; *CREBRF*; *PPP1R1B*; *DRC7*; *PLCD4*; *TH*; *TMEM95*; *CENPS*; *CTDNEP1*; *WBP2NL*; *NCAPH2*; *CLIC4*; *NR6A1*; *MEIOB*; *TCP1*; *MSH6*; *GNRH1*; *PRND*; *MAPK8IP2*; *C19H17*; *orf53*; *PSMA8*; *CCNE2*
Other items	22	*GNRHR*; *INSL3*; *KLC3*; *RNF4*; *SHBG*; *HBEGF*; *YBX2*; *IGF1*; *CDH1*; *GPX1*; *ITGA9*; *ITGAV*; *ITGB1*; *PIBF1*; *TCP11*; *CUBN*; *ACE*; *TFRC*; *INS*; *ADM*; *CCNA1*; *CDC25A*

Note: There are a total of 111 genes in the reproduction term, including developmental processes involved in reproduction, so only 31 genes that are not repeated are listed.

**Table 6:** Candidate genes for testis performance between immunocastration and control goats.

Gene	log2FC	*p*-Value	Kwithin	Go Enrich Item Number	Expression Level
*FGF9*	8.1290	0.0001	134.4660	5	Positive selection gene and DEseq
*FST*	5.2299	0.0234	144.7799	6	Positive selection gene and DEseq
*KIT*	−0.08929	0.9001	1180.2160	8	Positive selection gene and blue module
*TH*	5.8434	0.0024	1624.5921	5	Positive selection gene and DEseq
*TCP1*	−0.0973	0.7334	348.3450	2	Positive selection gene and red module
*PLEKHA1*	0.5119	0.3615	1771.9531	6	Positive selection gene and blue module
*TMEM119*	3.2132	0.0301	1084.8369	6	Positive selection gene and DEseq
*ESR1*	3.6093	0.0047	1669.9636	8	Positive selection gene and DEseq
*TIPARP*	0.4196	0.5861	1135.6891	5	Positive selection gene and blue module
*LEP*	−0.2711	0.9271	438.9352	8	Positive selection gene and blue module

## Data Availability

The original contributions presented in the study are included in the article; further inquiries can be directed to the corresponding authors.

## Supplementary Materials

The following supporting information can be downloaded at https://www.mdpi.com/article/10.3390/cells13010006/s1: Figure S1: Effect of PBK-asd DNA vaccine on testosterone of Yiling goats; Figure S2: Functional enrichment of DEG in buck groups. (A) The 10 items of GO enrichment in biological process. (B) The 10 items of KEGG pathway enrichment; Figure S3: The ME values in control group were correlated with phenotype traits; Figure S4: Gene significance (GS) across module and functional enrichment; Figure S5: Validation of transcriptome analysis results using qPCR analysis; Figure S6: The Neighbor-Joining phylogenetic tree of domestic and wild goats based on complete mitochondrial DNA sequences; Table S1: Primers used for quantitative reverse transcriptase PCR for goat; Table S2: Sample number and distribution of domestic and wild goats used in this study; Table S3: Summary of Yiling goat sample information and sequencing quality; Table S4: Information on the goat whole genome sequencing data used in this study; Table S5: Gene list intersection between RNA-seq and WGS analysis.

## Abbreviations

DEGs	Differentially expressed genes
ESR1	Estrogen receptor 1
ELISA	Enzyme-linked immunosorbent assay
FPKM	Fragments per kilobase of exon per million fragments mapped reads value
FDR	False discovery rate
FGF9	Fibroblast growth factor 9
FST	Follistatin
FSH	Follicle-stimulating hormone
GnRH	Gonadotropin-releasing hormone
GLM	General linear models
GO	Gene ontology
GS	Gene significant
HPG	Hypothalamic–pituitary–gland
HPT	Hypothalamic–pituitary–testis
KEGG	Kyoto Encyclopedia of Genes and Genomes
KIT	KIT proto-onco, receptor tyrosine kinase
KISS1	Kisspeptin
LH	Luteinizing hormone
LEP	Leptin
ME	Module eigengene
PBS	Phosphate buffer saline
PCA	Principal component analysis
PLEKHA1	Pleckstrin homology domain-containing A1
RNA-Seq	RNA transcriptome sequencing
TH	Tyrosine hydroxylase
TCP1	T-complex 1
TMEM119	Transmembrane protein 119
TIPARP	TCDD inducible poly (ADP-ribose) polymerase
WGS	Whole genome resequencing
WGCNA	Weighted gene co-expression network analysis
