# Supplementary material for: Multiple-Pathway Synergy Alters Steroidogenesis and Spermatogenesis in Response to an Immunocastration Vaccine in Goat
Source: Cells. 2023 Dec 20;13(1):6. doi: 10.3390/cells13010006 (PMC10778245; doi:10.3390/cells13010006)
Supplement: Supplementary file 1 [file cells-13-00006-s001.zip › cells-2711658-supplementary.pdf]

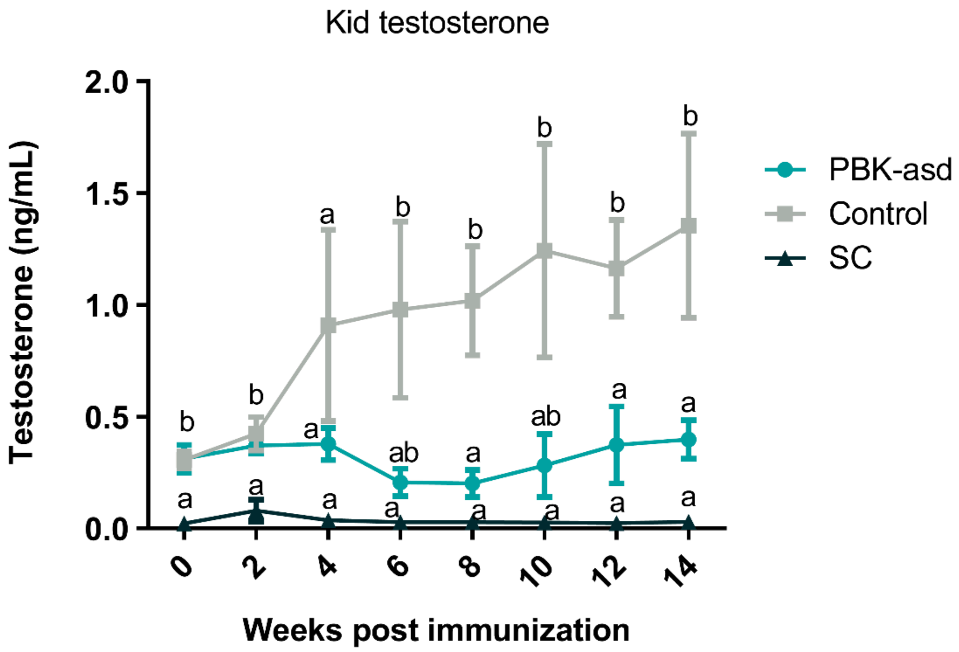

Supplementary Figure S1 Effect of PBK-asd DNA vaccine on testosterone of Yiling goats. The mean ± SEM serum testosterone hormone concentration in kid goats immunized with PBK-asd (n=5), surgically castrated (n=5), and control group (n=5), respectively. Different superscript letter among weeks within a treatment group indicates significant difference at $p < 0.05$.

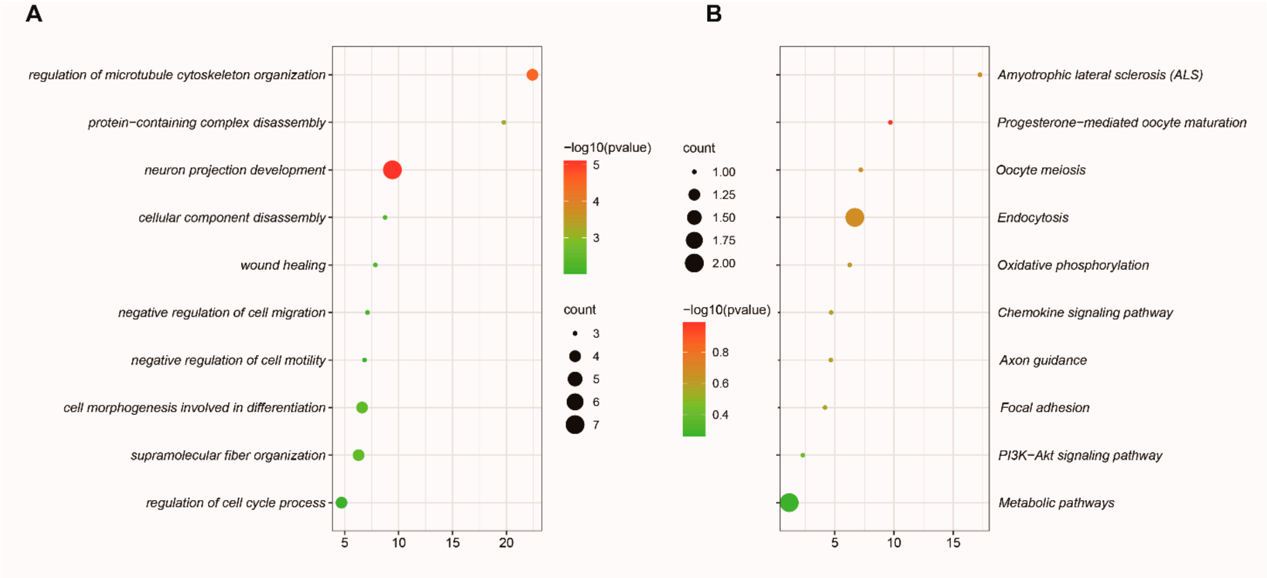

Supplementary Figure S2. Functional enrichment of DEG in buck groups. **A** The 10 items of GO enrichment in biological process. **B** The 10 items of KEGG pathway enrichment.

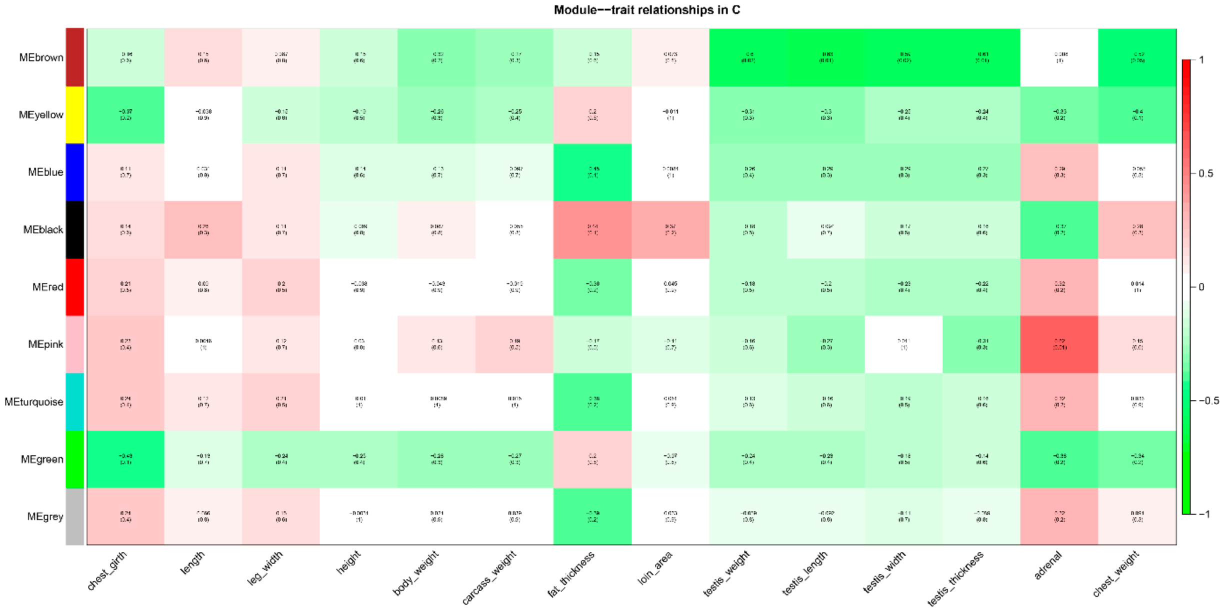

Supplementary Figure S3 The ME values in control group were correlated with phenotype traits. Each row in the table corresponds to a consensus module, and each column to a trait. Within each table cell, upper values represent correlation coefficients between ME and the variable, while lower values in brackets correspond to Student's asymptotic *p*-value.

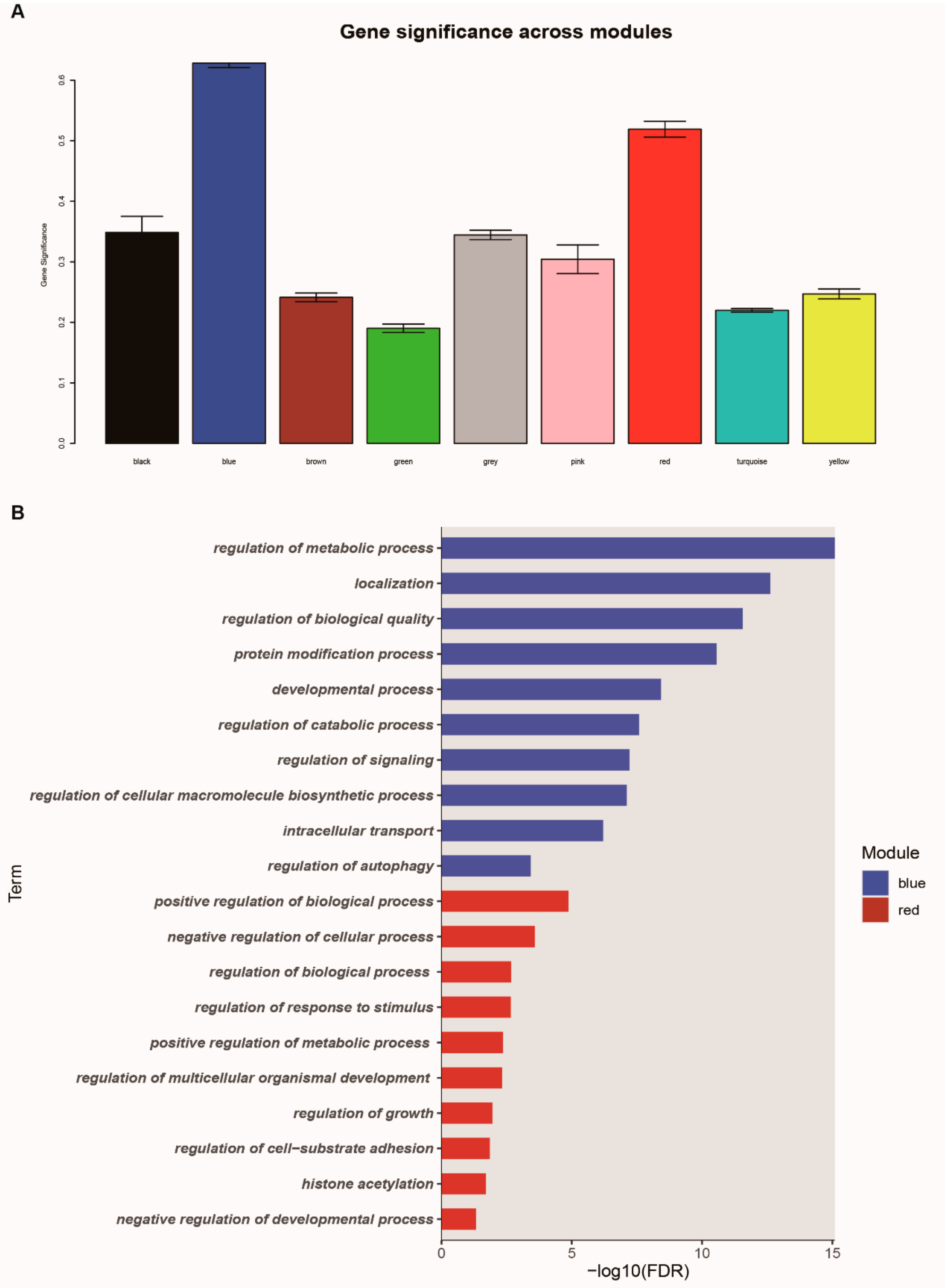

Supplementary Figure S4 Gene significance (GS) across module and functional enrichment. **A** GS of testis weight in each module. **B** Summary of Gene Ontology (GO)

analysis present under biological process of red and blue modules.

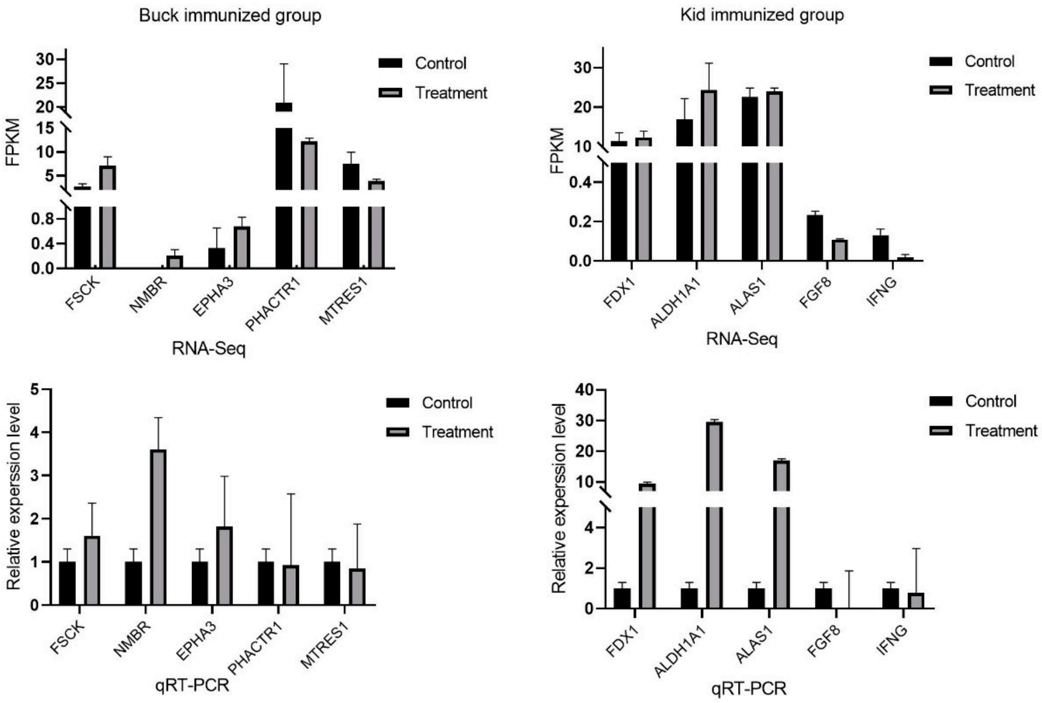

Supplementary Figure S5. Validation of transcriptome analysis results using qPCR analysis. The goat β-actin gene was used as reference gene. Data are shown as mean ± SD (n=3).

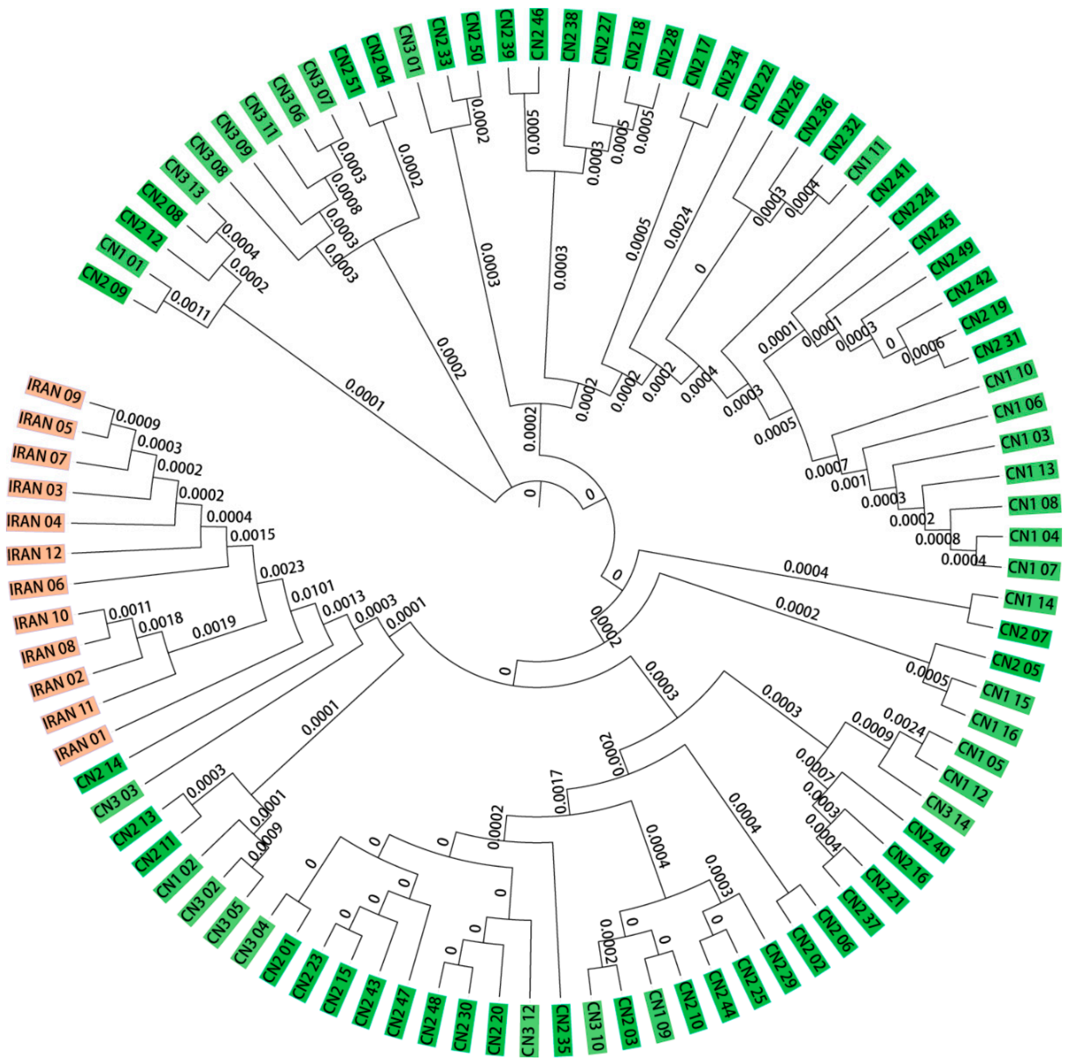

Supplementary Figure S6 The Neighbor-Joining phylogenetic tree of domestic and wild goats based on complete mitochondrial DNA sequences. Note: The green labels represent domestic goats and the orange labels represent wild goats. The values refer to genetic distance.

Supplement Table S1. Primers used for quantitative reverse transcriptase PCR for goat.

Gene	Gene Bank accession number	Primer sequence (5′-3′)	Product length (bp)	Annealing temperature (°C)
EPHA3	XM_005674802	F: GCTTGTCGACCGGGTTTCTA<br>R: GGGTCTTTGTCTGCTCGGAA	137	60
FCSK	XM_018061676	F: GGGGTTGATTGGACGGTCAT<br>R: TGGAAGACCTCAACGCTGTC	62	60
NMBR	XM_005684853	F: GCAGCTTGGATAATGGCAGC<br>R: TCATCCGTCTGAGGGTAGGG	55	60
PHACTR1	XM_018039147	F: GACGCTCAGGACTATGACCG<br>R: TCCTTCCGAATGGCAGCTTT	80	60
MTRES1	XM_018053106	F: GACTAGTGTCAGACTGCCCG<br>R: AGAGTCCAGTCCAGGCATCT	59	60
ALDH1A1	XM_005683753	F: CAAACCAGCAGAGCAAACCC<br>R: CGATCCTGTGAAGGCCACTT	166	60
FDX1	XM_018059813	F: ACAGATAGATCGCGGTTGGG<br>R: CATCAGACACGGCATCAGGT	88	60
ALAS1	XM_005695886	F: AACGTGAAGCTCATGAGGCA<br>R: AACGTGAAGCTCATGAGGCA	71	55
FGF8	XM_018041115	F: TCGAGTTCGAGGAGCTGAGA<br>R: TTCTGCAGGGCCGTGTAATT	138	60
IFNG	NM_001285682	F: TTCCGGTGGATGATCTGCAG<br>R: TACATTGATGCTCTCCGGCC	139	59

β-actin	NM_001314342.1	F: GTCACCAACTGGGACGACAT<br>R: CATCTTCTCACGGTTGGCCT	132	60

Supplement Table S2. Sample number and distribution of domestic and wild goats used in this study.

Population	Distribution	Sample number
**Domestic goats**		
Yiling goat	Hubei, China	35
Inner Mongolia cashmere goat	Inner Mongolia, China	3
Wuzhu Muqinbai goat	Inner Mongolia, China	1
Xinjiang goat	Xinjiang, China	2
Yimeng black goat	Shandong, China	1
Liaoning cashmere goat	Liaoning, China	1
Chengde goat (non-mountainous)	Hebei, China	1
Zhongwei goat	Ningxia, China	1
Jining gray goat	Shandong, China	1
Laiwu black goat	Shandong, China	1
Luliang black goat	Shanxi, China	1
Caidam goat	Qinghai, China	1
Jianza goat	Qinghai, China	6
Chengdu goat	Sichuan, China	1
Jianchang black goat	Sichuan, China	1
Shaannan white goat	Shaanxi, China	1
Anhui white goat	Anhui, China	1
Yaoshan white goat	Henan, China	1
Matou goat	Hubei, China	1

Xiangdong black goat	Hunan, China	1
Yvdong black goat	Chongqing, China	1
Maguan goat (non-mountainous)	Yunan, China	1
Guishan goat	Yunan, China	1
Guizhou black goat	Guizhou, China	1
Longlin goat	Guangxi, China	6
Leizhou goat	Hainan, China	1
Tibet goat	Tibet, China	8
**Wild goat**		
Iranian wild goat	Iran	12

Supplementary Table S3. Summary of Yiling goat sample information and sequencing quality.

Sample	Raw reads	Clean reads	Raw base (Gb)	Clean base (Gb)	Effective rate (%)	Fault tolerance rate (%)	Q20 (%)	Q30 (%)	GC content (%)
Yiling01	120796083	119775630	36.24	35.93	99.16	0.03	97.31	92.66	44.35
Yiling02	129627341	128525403	38.89	38.56	99.15	0.03	97.38	92.86	44.56
Yiling03	113911264	112878313	34.17	33.86	99.09	0.03	97.26	92.56	45.15
Yiling04	102886679	102076406	30.87	30.62	99.21	0.03	97.18	92.22	44.83
Yiling05	128846305	127781316	38.65	38.33	99.17	0.03	97.42	92.91	44.41
Yiling06	107339769	105893721	32.20	31.77	98.65	0.03	97.09	92.35	46.55
Yiling07	140889045	139189156	42.27	41.76	98.79	0.03	97.06	92.28	47.10
Yiling08	125236049	123809408	37.57	37.14	98.86	0.03	97.24	92.66	46.87
Yiling09	124264744	122896523	37.28	36.87	98.90	0.03	97.10	92.31	46.06
Yiling10	148525901	147056391	44.56	44.12	99.01	0.03	96.53	91.11	45.95
Yiling11	88474795	87884241	26.54	26.37	99.33	0.03	96.73	91.23	44.89
Yiling12	112723743	111682321	33.82	33.50	99.08	0.03	96.96	91.90	46.13
Yiling13	115615196	114507748	34.68	34.35	99.04	0.03	96.41	90.78	45.30

Yiling14	121107473	119891144	36.33	35.97	99.00	0.03	96.33	90.67	45.17
Yiling15	97054410	95787629	29.12	28.74	98.69	0.03	96.33	90.77	47.79
Yiling16	117771504	116949429	35.33	35.08	99.30	0.03	97.04	92.00	44.41
Yiling17	122730073	121887299	36.82	36.57	99.31	0.03	96.98	91.91	44.58
Yiling18	130319000	129279114	39.10	38.78	99.20	0.03	97.11	92.20	45.79
Yiling19	115051285	113348809	34.52	34.00	98.52	0.03	96.05	90.22	47.31
Yiling20	121221790	120256405	36.37	36.08	99.20	0.03	97.06	92.13	45.64
Yiling21	99238711	98272658	29.77	29.48	99.03	0.03	96.57	91.13	45.67
Yiling22	113162626	112139136	33.95	33.64	99.10	0.03	96.44	90.82	45.41
Yiling23	115482258	114164253	34.64	34.25	98.86	0.03	96.53	91.09	46.8
Yiling24	119504061	118632909	35.85	35.59	99.27	0.03	97.06	92.03	44.99
Yiling25	121270175	119963785	36.38	35.99	98.92	0.03	97.40	93.12	46.36
Yiling26	125062097	123999705	37.52	37.20	99.15	0.03	96.97	91.91	44.98
Yiling27	118220668	116802484	35.47	35.04	98.80	0.03	96.95	91.96	46.27
Yiling28	138511857	136908055	41.55	41.07	98.84	0.03	97.53	93.23	44.84
Yiling29	138858553	137480113	41.66	41.24	99.01	0.03	97.35	92.81	44.89
Yiling30	144182535	142576854	43.25	42.77	98.89	0.03	97.50	93.23	46.27

Yiling31	157495624	155312146	47.25	46.59	98.61	0.03	97.41	93.06	46.23
Yiling32	133828306	132883044	40.15	39.86	99.29	0.03	96.78	91.44	44.14
Yiling33	119329016	118346680	35.80	35.50	99.18	0.03	97.52	93.27	45.02
Yiling34	134688639	133415434	40.41	40.02	99.05	0.03	97.40	92.88	45.03
Yiling35	120983694	119889757	36.30	35.97	99.10	0.03	97.55	93.16	44.22

Supplementary Table S4. Information on the goat whole genome sequencing data used in this study.

Population	Name	Entry number	Size (Gb)	Population	Name	Entry number	Size (Gb)	Population	Name	Entry number	Size (Gb)
**Downloaded (wild)**				**Downloaded (domestic)**				**This study (domestic)**			
Iranian wild goat	IRCA01	ERR340348	32.4	Inner Mongolia cashmere goat	CN2_07	SRR5803189	41.1	Yiling goat	Yiling01	SRR16288442	35.9
	IRCA03	ERR340328	19.5		CN2_08	SRR5803186	39.9		Yiling02	SRR16288441	38.6
	IRCA04	ERR340343	21.7		CN2_09	SRR5803187	35.2		Yiling03	SRR16288430	33.9
	IRCA05	ERR340340	36.5	Shaannan white goat	CN2_10	SRR5803178	66.8		Yiling04	SRR16288419	30.6
	IRCA07	ERR340344	33.8	Wuzhu Muqinbai goat	CN2_11	SRR5803197	35.5		Yiling05	SRR16288413	38.3

	IRCA08	ERR340334	37.6	Xinjiang goat	CN2_12	SRR5803194	38.5		Yiling06	SRR16288412	31.8
	IRCA09	ERR340345	35.4		CN2_13	SRR5803181	35.8		Yiling07	SRR16288411	41.8
	IRCA10	ERR340338	34.2	Yaoshan white goat	CN2_14	SRR5803230	36.6		Yiling08	SRR16288410	37.1
	IRCA11	ERR340342	19.0	Yvdong black goat	CN2_50	SRR5803172	28.4		Yiling09	SRR16288409	36.9
	IRCA13	ERR340331	34.6	Zhongwei goat	CN2_51	SRR5803182	34.4		Yiling10	SRR16288408	44.1
	IRCA14	ERR340330	37.4	Chengde goat (non-mountainous)	CN3_01	SRR5803183	30.7		Yiling11	SRR16288440	26.4
	IRCA16	ERR340336	19.3	Jining gray goat	CN3_02	SRR5803177	35.4		Yiling12	SRR16288439	33.5
**Download (domestic)**				Laiwu black goat	CN3_03	SRR5803215	39.2		Yiling13	SRR16288438	34.4
Anhui white goat	CN1_01	SRR5803168	38.5	Leizhou goat	CN3_04	SRR5803231	40.9		Yiling14	SRR16288437	36.0

Caidam goat	CN1_02	SRR5803179	37.3	Liaoning cashmere goat	CN3_05	SRR5803180	40.8		Yiling15	SRR16288436	28.7
Jianza goat	CN1_03	SRR5803214	38.1	Longlin goat	CN3_06	SRR5803158	98.0		Yiling16	SRR16288435	35.1
	CN1_04	SRR5803213	39.4		CN3_07	SRR5803163	142.1		Yiling17	SRR16288434	36.6
	CN1_05	SRR5803212	39.1		CN3_08	SRR5803164	65.1		Yiling18	SRR16288433	38.8
	CN1_06	SRR5803205	40.0		CN3_09	SRR5803161	74.3		Yiling19	SRR16288432	34.0
	CN1_07	SRR5803209	38.3		CN3_10	SRR5803162	67.5		Yiling20	SRR16288431	36.1
	CN1_08	SRR5803229	39.2		CN3_11	SRR5803165	91.5		Yiling21	SRR16288429	29.5
Tibet goat	CN1_09	SRR5803196	38.7	Matou goat	CN3_12	SRR5803171	48.8		Yiling22	SRR16288428	33.6
	CN1_10	SRR5803195	38.6	Xiangdong black goat	CN3_13	SRR5803170	41.1		Yiling23	SRR16288427	34.3
	CN1_11	SRR5803203	38.6	Yimeng black goat	CN3_14	SRR5803206	40.0		Yiling24	SRR16288426	35.6
	CN1_12	SRR5803202	40.0						Yiling25	SRR16288425	36.0
	CN1_13	SRR5803201	39.1						Yiling26	SRR16288424	37.2
	CN1_14	SRR5803159	37.6						Yiling27	SRR16288423	35.0

	CN1_15	SRR5803160	50.5	Yiling28	SRR16288422	41.1
	CN1_16	SRR5803157	41.7	Yiling29	SRR16288421	41.2
Chengdu goat	CN2_01	SRR5803228	38.4	Yiling30	SRR16288420	42.8
Guishan goat	CN2_02	SRR5803184	35.8	Yiling31	SRR16288418	46.6
Guizhou black goat	CN2_03	SRR5803169	40.5	Yiling32	SRR16288417	39.9
JianChang black goat	CN2_04	SRR5803233	39.5	Yiling33	SRR16288416	35.5
Luliang black goat	CN2_05	SRR5803176	43.6	Yiling34	SRR16288415	40.0
Maguan goat without Angle	CN2_06	SRR5803185	59.5	Yiling35	SRR16288414	36.0

Supplementary Table S5. Gene list intersection between RNA-seq and WGS analysis.

Gene ID	Gene symbol	Ktotal	Kwithin	GO	log2FC (kid)	P value (kid)	Expression level
ENSCHIG00000020738	*FGF9*	10426.45	134.4661	5	8.129026592	0.0000678	Positive selection gene and DEseq
ENSCHIG00000019449	*SPIRE2*	10315.8	1036.03	3	-0.77240979	0.657520672	Positive selection gene and blue module
ENSCHIG00000022689	*HBEGF*	9942.656	924.4125	5	2.786824904	0.038100061	Positive selection gene and DEseq
ENSCHIG00000016855	*FST*	9561.124	144.7799	6	5.22995365	0.023462153	Positive selection gene and DEseq
ENSCHIG00000025706	/	9327.276	1289.693	/	0.315675843	0.528701321	Positive selection gene and blue module
ENSCHIG00000010514	*CNTFR*	9107.752	336.0197	3	2.573619922	0.105178386	Positive selection gene and red module

ENSCHIG00000019574	*SPINT2*	9024.999	1403.636	5	-0.41170049	0.494652897	Positive selection gene and blue module
ENSCHIG00000026162	*TH*	8320.337	1624.592	5	5.84341305	0.002422813	Positive selection gene and DEseq
ENSCHIG00000023115	*LFNG*	8074.776	398.6427	4	1.033803396	0.345061876	Positive selection gene and red module
ENSCHIG00000012744	*TMEM119*	8045.136	1084.837	6	3.213250281	0.03013029	Positive selection gene and DEseq
ENSCHIG00000014058	*ESR1*	7928.636	1669.964	8	3.609312617	0.004774435	Positive selection gene and DEseq
ENSCHIG00000012199	*WASHC5*	7103.418	1732.2	2	0.919589492	0.237829319	Positive selection gene and blue module
ENSCHIG00000021357	*TUBGCP6*	6822.656	416.1157	1	0.331270454	0.604250306	Positive selection gene and red module
ENSCHIG00000005289	*PLEKHA1*	6437.164	1771.953	6	0.511920671	0.361546324	Positive selection gene and blue module
ENSCHIG00000025200	/	6373.439	499.2954	/	1.137799076	0.638910976	Positive selection gene and red module
ENSCHIG00000009831	*NCAPH2*	6362.224	1428.043	3	-0.47835359	0.474208492	Positive selection gene and blue module
ENSCHIG00000026671	*KMT2B*	6098.937	528.9379	1	-0.13737029	0.787224148	Positive selection gene and red module
ENSCHIG00000006291	/	5922.709	507.9752	/	1.197973204	0.143033428	Positive selection gene and red module
ENSCHIG00000020968	*IGF2*	5452.408	1811.661	6	3.782886474	0.018219797	Positive selection gene and DEseq

ENSCHIG00000021157	*ITGA9*	3957.202	1689.496	2	0.631371522	0.408827811	Positive selection gene and blue module
ENSCHIG00000025994	*GREB1L*	2883.777	1642.253	4	0.90959272	0.360823303	Positive selection gene and blue module
ENSCHIG00000027016	*TFRC*	2795.096	1621.542	5	1.365451201	0.34793867	Positive selection gene and blue module
ENSCHIG00000017378	*NUPR1*	2785.277	659.6944	7	1.239973038	NA	Positive selection gene and red module
ENSCHIG00000008751	*BIRC6*	2223.435	1474.577	3	0.742441141	0.465405614	Positive selection gene and blue module
ENSCHIG00000016406	*GNRH1*	2049.511	1407.173	3	0.56643644	NA	Positive selection gene and blue module
ENSCHIG00000018721	*TIPARP*	1866.982	1135.689	5	0.419686389	0.586154972	Positive selection gene and blue module
ENSCHIG00000026354	*ASH1L*	1800.446	1287.815	7	0.714224707	0.563616413	Positive selection gene and blue module
ENSCHIG00000022287	*KIT*	1700.756	1180.216	8	-0.08929574	0.900112831	Positive selection gene and blue module

ENSCHIG00000021701	*UBAP2L*	1679.603	474.3126	2	-0.12160224	0.803507938	Positive selection gene and red module
ENSCHIG00000012304	*HERC4*	1240.99	959.4366	1	0.306485904	0.570249446	Positive selection gene and blue module
ENSCHIG00000016503	*TCP1*	1071.71	348.345	2	-0.09735723	0.733437526	Positive selection gene and red module
ENSCHIG00000010015	*MYORG*	714.8215	278.8131	2	-0.29566244	0.493457543	Positive selection gene and blue module
ENSCHIG00000019616	*LEP*	639.2835	438.9353	8	-0.27110208	0.927132382	Positive selection gene and blue module
